# Corilagin Ameliorates Con A-Induced Hepatic Injury by Restricting M1 Macrophage Polarization

**DOI:** 10.3389/fimmu.2021.807509

**Published:** 2022-01-13

**Authors:** Fenglian Yan, Dalei Cheng, Haiyan Wang, Min Gao, Junfeng Zhang, Hongyan Cheng, Changying Wang, Hui Zhang, Huabao Xiong

**Affiliations:** ^1^ Institute of Immunology and Molecular Medicine, Jining Medical University, Jining, China; ^2^ Jining Key Laboratory of Immunology, Jining Medical University, Jining, China; ^3^ Cheeloo College of Medicine, Shandong University, Jinan, China; ^4^ Clinical Laboratory, Jining First People’s Hospital, Jining, China

**Keywords:** corilagin, concanavalin A, hepatic injury, macrophage, inflammation

## Abstract

Immune-mediated hepatic injury plays a key role in the initiation and pathogenesis of diverse liver diseases. However, treatment choice for immune-mediated hepatic injury remains limited. Corilagin, a natural ellagitannin extracted from various traditional Chinese medicines, has been demonstrated to exhibit multiple pharmacological activities, such as anti-inflammatory, anti-tumor, and hepatoprotective properties. The present study aimed to investigate the effects of corilagin on immune-mediated hepatic injury using a murine model of concanavalin A (Con A)-induced hepatitis, which is well-characterized to study acute immune-mediated hepatitis. Herein, mice were administered corilagin (25 mg/kg) intraperitoneally twice at 12 h intervals, and 1 h later, the mice were challenged with Con A (20 mg/kg body weight); serum and liver samples were collected after 12 h. The results showed that corilagin significantly increased the survival of mice and reduced serum alanine transaminase (ALT) and aspartate aminotransferase (AST) levels. In addition, corilagin markedly improved histopathological damage, hepatocyte apoptosis, and oxidative stress in the liver. The activation of M1 macrophages in the hepatic mononuclear cells was also significantly reduced compared with that in the control group. The expression of M1 macrophage-associated proinflammatory cytokines and genes, including interleukin (IL)-6, IL-12, and inducible nitric oxide synthase (iNOS), was also decreased after corilagin treatment. Finally, the results demonstrated that corilagin regulated macrophage polarization by modulating the mitogen-activated protein kinases (MAPK), nuclear factor (NF)-κB, and interferon regulatory factor (IRF) signaling pathways. Thus, the findings indicate that corilagin protects mice from Con A-induced immune-mediated hepatic injury by limiting M1 macrophage activation *via* the MAPK, NF-κB, and IRF signaling pathways, suggesting corilagin as a possible treatment choice for immune-mediated hepatic injury.

## Introduction

The liver is an important immune organ of the human body and contains numerous innate and adaptive immune cells (1, 2). Liver diseases, including hepatitis, liver fibrosis, and hepatic carcinoma, pose serious challenges to global public health (3). Immune-mediated hepatic injury is a crucial step in the progression of liver diseases (4, 5). Concanavalin A (Con A)-induced hepatic injury murine model is the most commonly used for acute immune-mediated hepatitis due to pathophysiological characteristics similar to those of clinical autoimmune hepatitis (AIH) and viral hepatitis (6, 7). Accumulating evidence suggests that the initiation and development of liver damage in this model is tightly controlled by interactions between multiple cell types and cytokines. Immune cells involved in this model mainly include T cells, natural killer (NK) cells, natural killer T (NKT) cells, and Kupffer cells (liver macrophages) (4, 6–9).

As T lymphocyte mitogen, Con A primarily leads to recruitment and activation of T cells after intravenous injection, which induces hepatotoxic cytokines such as IFN-γ, TNF-α, IL-4, IL-5, and eventually lead to liver injury (7, 10). NKT cells, which account for a large proportion in mice liver, can aggravate inflammation and liver injury by the apoptosis related factor ligand (FasL), perforin, granzyme and inflammatory cytokines (11, 12). Accumulating studies have shown that NK cells also play an important role in this model. Wang et al. found that activation of NK cells by poly I:C had an inhibitory effect on Con A-induced liver injury *via* downregulating NKT and T cells and inhibiting IFN-γ and IL-4 (13). But, Takeda K et al. showed that depletion of NK cells did not significantly affect Con A-induced liver injury (14). However, the exact contributions of immune cells to immune-mediated hepatic injury is still not fully understood.

Liver macrophages account for approximately 20% of liver immune cells, including resident Kupffer cells and bone marrow-derived macrophages under pathological conditions. These cells play a critical role in the detection, capture, and clearance of pathogens (2). Therefore, they are regarded as important mediators of liver homeostasis (15). Studies have demonstrated that the depletion of Kupffer cells using gadolinium chloride could completely prevent Con A-induced liver injury and the release of cytokines (16, 17). Using Kupffer cell-removed mice (obtained through administration of liposome-encapsulated dichloromethylene bisphosphonate or clodronate liposomes), Hatano et al. and Schumann et al. also showed that Kupffer cells contribute to Con A-induced liver injury by producing macrophage inflammatory protein (MIP)-2, keratinocyte chemoattractant (KC), IL-4, and tumor necrosis factor-α (TNF-α) (18, 19). Nakashima et al. found that the production of superoxide and reactive oxygen species (ROS) by activated macrophages is the main effector in the development of Con A-induced hepatitis (16). In general, all these studies demonstrated that liver macrophages play a pivotal role in Con A-induced hepatic injury by producing pro-inflammatory cytokines and superoxide (20).

Corilagin (C_27_H_22_O_18_), an ellagitannin belonging to the tannin family, is one of the major bioactive compounds in many medicinal plants (21). It was first extracted from *Caesalpinia coriaria* by Schmidt et al. (22) in 1951 and is reported to have various biological and pharmacological activities, including hepatoprotective, anti-inflammatory, anti-microbial, anti-oxidant, anti-hypertensive, anti-diabetic, and anti-tumor properties (23). It can inhibit the growth of multiple cancer cells by promoting apoptosis and inhibiting cell proliferation (21). Because of its low cytotoxicity in normal tissues and cells, it is regarded as a candidate molecule for the treatment of cancer. The hepatoprotective effects of corilagin have been reported in some liver-related diseases, such as liver fibrosis, drug-induced liver damage, and hepatic carcinoma. Du et al. demonstrated that corilagin can ameliorate schistosomiasis hepatic fibrosis by regulating the IL-13, GATA3, and miR21/smad7/extracellular regulated protein kinases (ERK) signaling pathways (24–27). Lv et al. and Liu et al. indicated that corilagin could alleviate acetaminophen-induced hepatotoxicity by regulating the MAPK, NF-κB, and AMP-activated protein kinase (AMPK)/glycogen synthase kinase 3 β (GSK3β)- Nuclear factor erythroid 2-related factor 2 (Nrf2) signaling pathways (28, 29). Joshi et al. suggested that corilagin could protect Sprague-Dawley rats against D-GalN/LPS-induced liver injury by suppressing oxidative stress and apoptosis (30). Another study demonstrated that corilagin has protective effects on hemorrhagic shock-induced liver injury through the regulation of the Akt-dependent pathway (31). Corilagin blocks hepatitis C virus (HCV) replication and modulates oxidative stress, thereby reducing liver damage (32). However, the precise mechanism underlying the hepatoprotective effects of corilagin is still incompletely understood. In particular, how the immune cells involved in this process require further investigation. Therefore, in this study, we aimed to explore the role of macrophages in the protective effect of corilagin in the liver using a Con A-induced hepatic injury model.

## Materials and Methods

### Reagents

Con A (Cat:C2010) was purchased from Sigma-Aldrich (St. Louis, MO, USA). Corilagin (purity ≈ 99.95%) was obtained from MCE (NJ, USA). Myeloperoxidase (MPO), malondialdehyde (MDA), superoxide dismutase (SOD) test kits were obtained from the Nanjing Jiancheng Bioengineering Institute (Nanjing, China). IL-6, IL-12, and TNF-α ELISA kits, and flow cytometric analysis related antibodies were supported by BioLegend (San Diego, CA, USA). Quantitative reverse transcription-polymerase chain reaction (qRT-PCR)-related reagents were provided by Vazyme (Nanjing, China), including AceQ qPCR SYBR Green Master Mix (Cat: Q111) and HiScript III RT Super Mix for qPCR (Cat: R323). Antibodies against p-JNK (Cat: 4668), JNK (Cat: 9252), p-ERK (Cat: 4370), ERK (Cat: 4695), p-p38 (Cat: 4511), p38 (Cat: 8690), p65 (Cat: 8242), and p-p65 (Cat: 3033) were purchased from Cell Signaling (Boston, MA, USA). IRF5 antibody (Cat: ab181553) was purchased from Abcam (Cambridge, MA). Anti-β-actin (Cat: AA128), HRP-labeled goat anti-rabbit IgG (Cat: A0208), and HRP-labeled goat anti-mouse IgG (Cat: A0216) antibodies were obtained from Beyotime Biotechnology (Shanghai, China).

### Animals and Mouse Model

**Figure 1:**
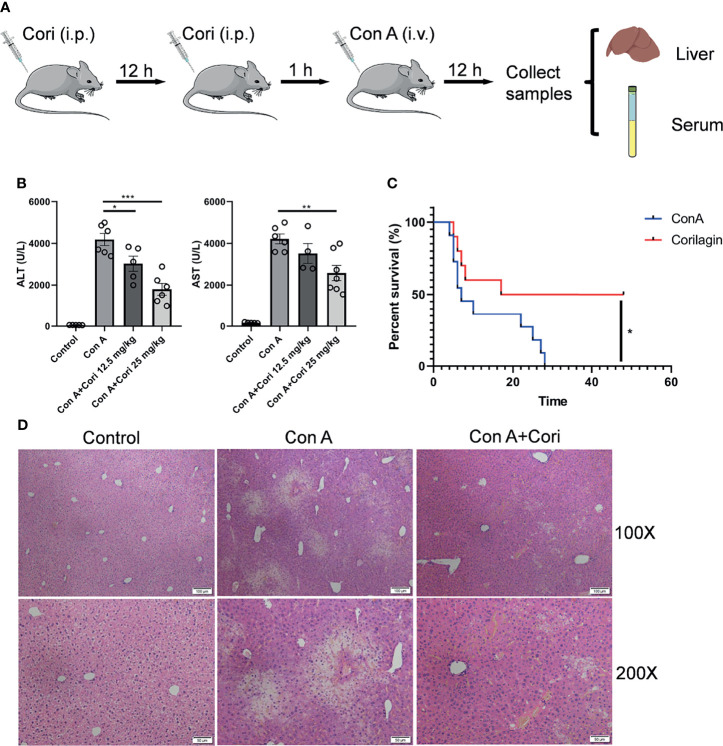
Corilagin ameliorates Con A-induced hepatic injury. Mice were administered with corilagin (12.5, 25 mg/kg) intraperitoneally twice at time intervals of 12 h. After 1 h, mice were challenged with Con A (20 mg/kg body weight). After 12 h, serum and livers were collected. **(A)** Flowchart for drug administration. **(B)** Serum alanine aminotransferase (ALT) and aspartate aminotransferase (AST) levels. **(C)** Survival experiments performed with mice treated with a lethal dose of Con A (25 mg/kg body weight; n = 10). **(D)** Liver sections stained with hematoxylin and eosin (H&E). Original magnification, 100× and 200×. *p < 0.05, **p < 0.01, ***p < 0.001.

Male C57BL/6J mice (weight: 18–22 g, age: 6–8 weeks) were provided by the Peng Yue Experimental Animal Breeding Company (Jinan, China). All experiments involving animals were conducted according to the guidelines for the care and use of laboratory animals approved by the Animal Care Committee of Jining Medical University. C57BL/6J mice were randomly divided into different groups according to experimental design (control groups n=5, experiment groups n=7). The control and experimental groups were administered solvent or corilagin (12.5, 25 mg/kg body weight) intraperitoneally twice at time intervals of 12 h. After 1 h, the mice were challenged with Con A (20 mg/kg body weight). After 12 h, serum and liver samples were collected. The flowchart for drug administration was showed in **Figure 1A**. Survival experiments were performed in mice treated with a lethal dose of Con A (25 mg/kg body weight).

### Biochemical Index Assays

Serum alanine transaminase (ALT) and aspartate aminotransferase (AST) levels were measured with a multiple biochemical analyzer (Cobas 8000, Roche, Basel, Switzerland) after 10× dilution with physiological saline. SOD, MDA, and MPO levels in the hepatic homogenates were measured according to the manufacturer’s instructions.

### Histopathological Analysis

The collected liver tissues were fixed with 4% formalin immediately and then embedded in paraffin. After cutting into 5 μm-thick sections, the tissues were stained with hematoxylin-eosin (H&E) and observed using an optical light microscope. *In situ* terminal deoxynucleotidyl transferase-mediated uridine triphosphate nick-end labeling (TUNEL) assays (Cat: 11684817910, Roche) were performed following the manufacturer’s protocol.

### Measurement of Serum Cytokine Levels by ELISA

Serum cytokine levels, including those of IL-6, IL-12, and TNF-α, were measured using ELISA kits (BioLegend). The standard procedure was performed according to the manufacturer’s instructions.

### Isolation of Hepatic Mononuclear Cells (HMNCs)

Isolation of hepatic mononuclear cells was performed using a method established previously with minor modifications (33). The mice were exposed to anesthesia. After cutting a slot in the auricula dextra, the livers were perfused with 20 ml PBS from the left ventricle. We collected and ground the liver and filtered the hepatic homogenates using a 200-mesh stainless steel screen. The harvested hepatic homogenates were centrifuged at 1000 × *g* for 10 min. The pellets were resuspended in 50 ml PBS and centrifuged at 20 × *g* for 5 min to remove tissue clumps. The supernatants were centrifuged at 700 × *g* for 8 min. The pellets were resuspended in 3 ml of 40% Percoll, which was loaded onto 70% Percoll. After centrifuged at 700 × *g* for 30 minutes. HMNC was obtained from the mesophase and washed twice in PBS.

### Preparation of Bone Marrow-Derived Macrophages (BMDMs)

C57BL/6 mice were prepared on a clean bench, and the bone marrow from the tibias and femurs was rinsed. The obtained bone marrow cells were filtered through a 200-mesh stainless steel screen. The red blood cells in the bone marrow were lysed with red blood cell lysis solution. The remaining cells were cultured in complete Dulbecco’s modified Eagle medium (DMEM, Invitrogen, Shanghai, China) supplemented with GM-CSF (10 ng/ml; PeproTech, NJ, USA) for 7 days.

### Flow Cytometry Analysis

The HMNCs and BMDMs obtained were processed into single-cell suspensions with PBS for surface staining and labeled with optical antibodies for 30 min at 4°C. After washing with PBS, the cells were analyzed with a BD FACS verse flow cytometer (Becton Dickinson, NJ, USA).

### Cell Viability Assay

The BMDMs prepared were seeded onto a 96-well plate (Corning, NY, USA) and treated with different concentrations of corilagin (1, 5, 10, 20 μg/ml) for 2 h (5×10^4^ cells/well). Next, the cells were stimulated using lipopolysaccharide (LPS) (200 ng/ml) and interferon γ (IFN-γ) (10 ng/ml). After 24 h, cell viability was measured using the CellTiter-Lumi luminescent cell viability assay kit (Beyotime, Shanghai, China) according to the manufacturer’s guidelines.

### Quantitative Real-Time PCR Analysis

Total RNA was extracted from liver tissues and *in vitro* cultured cells using RNAiso Plus reagent (TaKaRa, Japan) after homogenization with a tissue homogenizer (KZ-III, Servicebio, Wuhan, China). cDNA was amplified from 1 μg total RNA using HiScript III RT Super Mix for qPCR (Vazyme). Real-time PCR was performed with a Light Cycler 480 system (Roche), using AceQ qPCR SYBR Green Master Mix (Vazyme). The specific primers used were as follows, IL-6 forward 5’-CCAGAAACCGCTATGAAGTTCCT-3’ and reverse 5’-CACCAGCATCAGTCCCAAGA-3’, IL-12 forward 5’-AGACATGGAGTCATAGGCTCTG-3’ and reverse 5’-CCATTTTCCTTCTTGTGGAGCA-3’, TNF-α forward 5’-GCCACCACGCTCTTCTGTCT-3’ and reverse 5’-GGTCTGGGCCATAGAACTGATG-3’, iNOS forward 5’-CTGCAGCACTTGGATCAGGAACCTG-3’ and reverse 5’-GGAGTAGCCTGTGTGCACCTGGAA-3’, IL-1β forward 5’-TGGACCTTCCAGGATGAGGACA-3’ and reverse 5’-GTTCATCTCGGAGCCTGTAGTG-3’, glyceraldehyde 3-phosphate dehydrogenase (GAPDH) forward 5’-AACGACCCCTTCATTGAC-3’ and reverse 5’-TCCACGACATACTCAGCAC-3’. The specific steps were performed according to the manufacturer’s instructions.

### Western Blotting

The total proteins of cultured macrophages were lysed with RIPA Lysis Buffer (Cat: P0013B, Beyotime) containing a protease inhibitor cocktail (Sigma-Aldrich) and PMSF (Beyotime). The concentrations of these samples were detected using an enhanced BCA protein assay kit (Cat: P0010, Beyotime). Then, the lysates were loaded on a 10% sodium dodecyl sulfate polyacrylamide gel. The proteins on the gel were transferred to a 0.45-μm PVDF membrane (Sigma-Aldrich) using the wetting transfer method. The membranes were blocked with 3% BSA (w/v) for approximately 1 h. Next, the membranes were incubated with specific primary antibodies overnight in a 2–8°C freezer. After washing three times with TBST the following day, the membranes were probed with HRP-conjugated secondary antibodies for approximately 2 h at 20–25°C. Finally, the Pierce ECL Western Blotting Substrate (Thermo Fisher Scientific, Waltham, MA, USA) was used to detect the immunoreactive signals on the membrane using an Amersham Imager 600 chemiluminescence instrument (General Electric Company, CT, USA).

### Macrophage Adoptive Transfer

BMDMs were treated with corilagin (10 μg/ml); 3 h later, these cells were collected and 2×10^6^ cells/mouse were injected intraperitoneally. After 12 h, the mice were challenged with Con A (20 mg/kg body weight). Serum and liver tissues were collected 12 h after the challenge.

### Statistical Analysis

All the results were analyzed using the GraphPad Prism software (version 8.0) and shown as the mean ± standard error of mean (SEM). One-way analysis of variance (ANOVA) and Student’s t-test were used to analyze the differences between multiple groups and two groups, respectively. When the p-values were less than 0.05 (*), 0.01 (**), and/or 0.001(***), the difference was considered statistically significant.

## Results

### Corilagin Improved Con A-Induced Hepatic Injury

It has been reported that corilagin elicits the hepatoprotective effects in liver related diseases, such as acetaminophen-induced hepatotoxicity, hepatic fibrosis, hepatocellular carcinoma and so on. However, immune-mediated hepatic injury is a crucial step in progression of those liver diseases (4, 5). The immune cells involved in hepatoprotective effects of corilagin were still unclear. Therefore, Con A induced liver injury mouse model was used in our study. To investigate the effects of corilagin on Con A-induced injury, different concentrations of this substance were administered to a mouse model. C57BL/6 mice were randomly divided into 4 groups. The experimental groups were administered corilagin (12.5, 25 mg/kg body weight) intraperitoneally twice at time intervals of 12 h. The flowchart for drug administration was showed in **Figure 1A**. ALT and AST levels were routinely quantified to assess liver damage. Therefore, we compared ALT and AST levels between the control and experimental groups after Con A challenge for 12 h. The results showed that the levels of ALT and AST were significantly decreased in the corilagin treatment groups, particularly in the 25 mg/kg corilagin treatment group (**Figure 1B**). Liver hemorrhage also improved after corilagin treatment, as shown in **Supplementary Figure 1**. Therefore, subsequent experiments were conducted at this concentration. After challenge with a lethal dose of Con A, the survival rate of the corilagin-treated group was markedly improved compared with that of the control group, as shown in **Figure 1C**. H&E staining results demonstrated noticeably disturbed architecture in the liver tissue of the Con A-treated group, including hepatocyte necrosis, hemorrhage, and inflammatory cell infiltration, and the damage was notably improved in the corilagin-pretreated group (**Figure 1D**). Thus, the results suggest that corilagin alleviated Con A-induced hepatic injury. To investigate if corilagin alone has any effect on the liver and what happens during corilagin pretreatment process, we set a control experiment. Mice were administered corilagin (25 mg/kg body weight) intraperitoneally twice at time intervals of 12 h. 13 h later, serum and liver samples were collected (n=10). The flowchart for corilagin administration was presented in **Supplementary Figure 2A**. As shown in **Supplementary Figure 2B**, the ALT and AST results indicated that corilagin alone had no side effects on the mice.

### Corilagin Attenuated Con A-Induced Oxidative Stress and Hepatocyte Apoptosis

**Figure 2:**
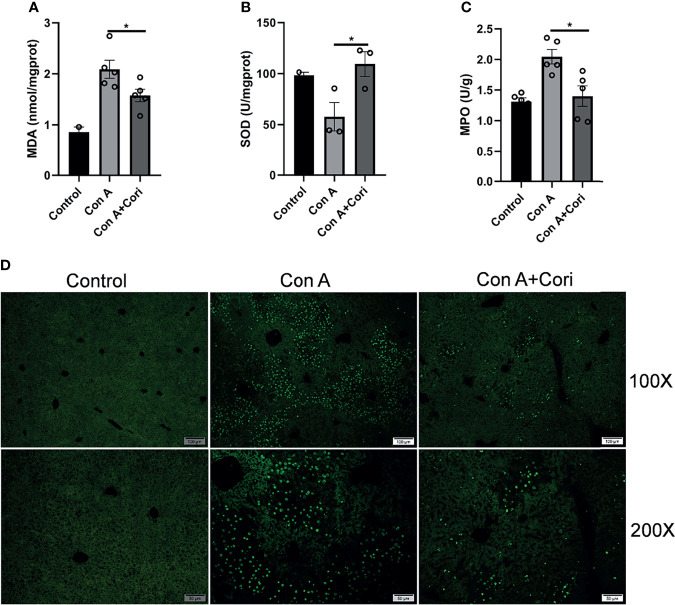
Corilagin attenuates Con A-induced oxidative stress and hepatocyte apoptosis in the liver tissue. Mice were administered corilagin (25 mg/kg) intraperitoneally twice at time intervals of 12 h. After 1 h, mice were challenged with Con A (20 mg/kg body weight). Liver tissues were collected 12 h later. Effects of corilagin on liver **(A)** malondialdehyde (MDA), **(B)** Superoxide dismutase (SOD), and **(C)** myeloperoxidase (MPO). **(D)** Liver sections stained with TUNEL assay. Original magnification, 100× and 200×. *p < 0.05.

Oxidative stress has been demonstrated to play an important role in Con A-induced immune-mediated liver injury. Therefore, we measured MDA level and SOD activity after corilagin treatment. As previously reported, the level of MDA increased and SOD activity decreased in the Con A group (34, 35). As shown in **Figures 2A, B**, corilagin treatment significantly reduced the MDA level and increased the activity of SOD compared with the Con A group. We also examined MPO levels in the liver, which indicate neutrophil infiltration. MPO levels in the corilagin-treated group were significantly reduced compared with those in the Con A-treated group (**Figure 2C**). These results are consistent with those of other studies on the antioxidant effects of corilagin on other mouse models, such as the acetaminophen-induced hepatotoxicity model and ischemia/reperfusion-induced acute lung injury model (28, 29, 36). Previous studies have shown that necrotic hepatocyte death induced by Con A is accompanied by massive apoptosis (35, 37). Oxidative stress is one of the factors that induce apoptosis (38). Therefore, we examined hepatocyte apoptosis using the TUNEL assay. The results showed that corilagin treatment markedly attenuated hepatocyte apoptosis induced by Con A (**Figure 2D**). This result is also consistent with other studies suggest that corilagin has anti-apoptotic activity in an ischemia/reperfusion-induced acute lung injury model (36).

### Corilagin Protected Against Con A-Induced Liver Injury by Regulating M1 Macrophage Activation

**Figure 3:**
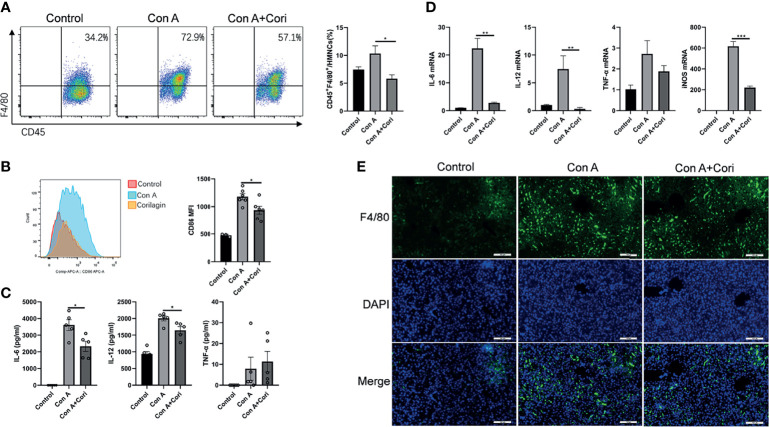
Corilagin protects against Con A-induced liver injury by regulating the activation of M1 macrophages. Mice were administered corilagin (25 mg/kg) intraperitoneally twice at time intervals of 12 h. After 1 h, mice were challenged with Con A (20 mg/kg body weight). Liver tissues were collected 12 h later. **(A)** Detection of the macrophage ratio in mouse hepatic mononuclear cells (HMNCs) by flow cytometry. **(B)** CD86 mean fluorescence intensity (MFI). **(C)** Inflammatory cytokines interleukin (IL)-6, IL-12, and tumor necrosis factor-α (TNF-α) in the serum measured by ELISA. **(D)** IL-6, IL-12, TNF-α, and inducible nitric oxide synthase (iNOS) levels in the liver tissue measured by qRT-PCR. **(E)** Macrophage infiltration in the liver tissue 200×. *p < 0.05, **p < 0.01, ***p < 0.001.

Considering that macrophage depletion prevents mice from developing Con A hepatitis (16, 18, 19), in the present study, we mainly focused on the effects of corilagin on macrophages. First, we determined the proportion and CD86 mean fluorescence intensity (MFI) of macrophages in the liver by flow cytometry. The proportion of macrophage cells (F4/80^+^ cells) and CD86 MFI increased in the Con A group. However, this increase was significantly reduced by corilagin treatment (**Figures 3A, B**). The detailed gating strategies were presented in **Supplementary Figure 3**. To further demonstrate the effects of corilagin on macrophages, we examined macrophage infiltration in liver tissue using an immunofluorescence assay (**Figure 3E**). The results showed that treatment with corilagin effectively protected against Con A-induced hepatic injury by inhibiting M1 macrophage activation. We measured the expression of M1 macrophage-related inflammatory cytokines and genes, including IL-6, IL-12, TNF-α, and iNOS, in serum and liver tissue using ELISA and quantitative real-time PCR, respectively. As shown in **Figures 3C, D**, corilagin decreased IL-6 and IL-12 levels in the serum and liver tissues. Furthermore, the expression of iNOS, which is regarded as the signature gene of M1 macrophages, was significantly inhibited by corilagin treatment. The results indicated that corilagin could suppress M1 macrophage activation to protect against liver injury induced by Con A.

In addition to macrophages, other immune cells including T cells, NK cells, NKT cells, and MDSCs (4, 6–9) are involved in this model. We analyzed the proportion of T, NK, NKT cells, and MDSCs in the liver using flow cytometry. The results are consistent with those of previous studies. The proportion of activated T, NK, and NKT cells significantly increased after Con A challenge. However, corilagin treatment significantly reduced the activation of NKT and T cells (see **Supplementary Figure 4**). The activation of NK cells was comparable with that of the Con A group. In agreement with the finding of Diao et al. (9), we also found that MDSCs were expanded after the Con A challenge. Interestingly, after corilagin treatment, the proportion of MDSCs was further increased. Our future studies will investigate the effect of corilagin on these cells. We also detected the effect of corilagin treatment alone on the liver lymphocyte, including macrophage, NK, NKT, T, MDSCs cells and the activated NK, NKT, T cells. Mice were treatment as indicated in **Supplementary Figure 2**. The results were showed in **Supplementary Figures 5, 6**. And the data suggested that corilagin pretreatment alone has no effect on mouse liver lymphocyte.

**Figure 4:**
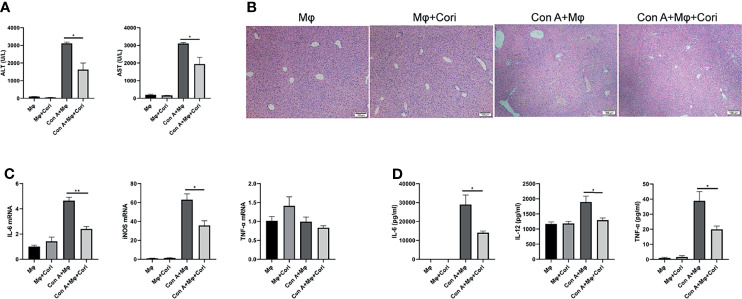
Corilagin treated macrophage protects against Con A-induced liver injury in an adoptive transfer experiment. Macrophage cultured *in vitro* were treated with corilagin (10 μg/ml); 3 h later these cells were collected and injected into mice intraperitoneally. After 12 h, mice were challenged with Con A (20 mg/kg body weight). Liver tissues were collected 12 h later. **(A)** Serum ALT and AST levels; **(B)** H&E staining of liver tissues. Original magnification, 100×. **(C)** IL-6, iNOS, and TNF-α expression in the liver detected by qRT-PCR. **(D)** IL-6, IL-12, TNF-α levels in serum detected by ELISA. *p < 0.05, **p < 0.01.

We also performed adoptive transfer experiments *in vivo* to verify the protective effect of corilagin-treated macrophages. The results showed that ALT, AST levels and liver hemorrhage were significantly decreased after adoptive transfer with corilagin-treated macrophages (**Figure 4A** and **Supplementary Figure 7**). As shown in **Figure 4B**, the histopathological damage to liver tissues was also significantly improved after adoptive transfer with corilagin-treated macrophages. Moreover, the expression of M1 macrophage signature genes, including *iNOS* and *IL-6*, were markedly reduced in the liver (**Figure 4C**). The release of proinflammatory cytokines (IL-6, IL-12, and TNF-α) into the serum were also markedly reduced (**Figure 4D**).

### Corilagin Inhibited M1 Macrophage Activation and Inflammation Cytokine Release *In Vitro*

**Figure 5:**
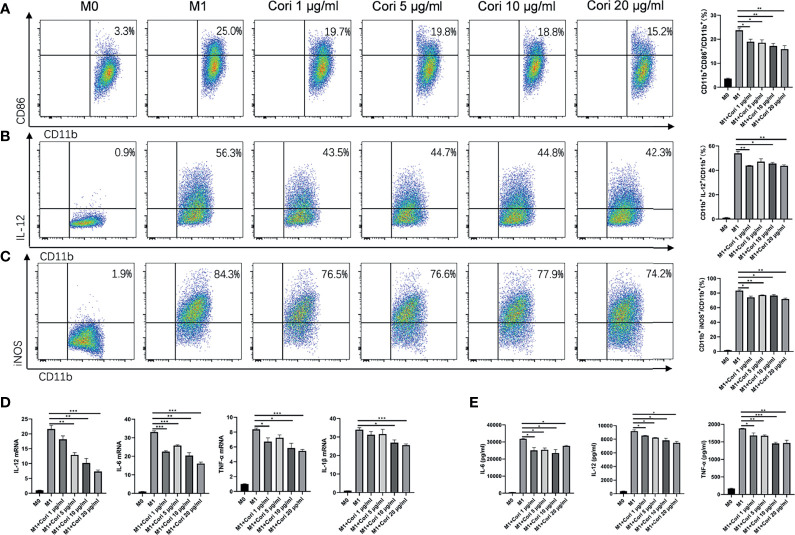
Corilagin reduces the activation of M1 macrophages and inhibits inflammatory cytokine release *in vitro*. BMDMs were treated with corilagin (1, 5, 10, or 20 μg/ml) for 2 h, followed by stimulation with lipopolysaccharide (LPS) and interferon γ (IFN-γ). After 24 h, the supernatant and cells were collected. **(A)** Proportion of CD86^+^ cells. **(B)** Proportion of IL-12^+^ cells. **(C)** Proportion of iNOS^+^ cells. **(D)** mRNA levels of IL-12, IL-6, TNF-α, and IL-1β. **(E)** Protein levels of IL-12, IL-6, and TNF-α in the cell culture supernatant. *p < 0.05, **p < 0.01, ***p < 0.001.

Next, we investigated the effects of corilagin on M1 macrophages *in vitro*. BMDMs were treated with corilagin (1, 5, 10, and 20 μg/ml) for 2 h followed by stimulation with IFN-γ and LPS. After 24 h, we determined the cytotoxicity of corilagin on macrophages using the CellTiter-Lumi luminescent cell viability assay kit. The results indicated that corilagin had no cytotoxicity on macrophages even at the maximum concentration (20 μg/ml; **Supplementary Figure 8**). We also collected the cells to detect the proportion of CD86^+^, iNOS^+^, and IL-12^+^ cells, which are indicative of M1 macrophages (**Figures 5A–C**). The detailed gating strategies were presented in **Supplementary Figure 9**. The results showed that corilagin treatment markedly reduced the proportion of these cells. We then detected the expression of macrophage-related inflammatory cytokines (IL-6, IL-12, TNF-α, and IL-1β) using qRT-PCR and ELISA. The results, presented in **Figures 5D, E**, suggest that corilagin treatment significantly blocked the release of inflammatory cytokines from M1 macrophages.

### Corilagin Inhibited M1 Macrophage Activation by Inhibiting the MAPK, NF-κB, and IRF Signaling Pathways

**Figure 6:**
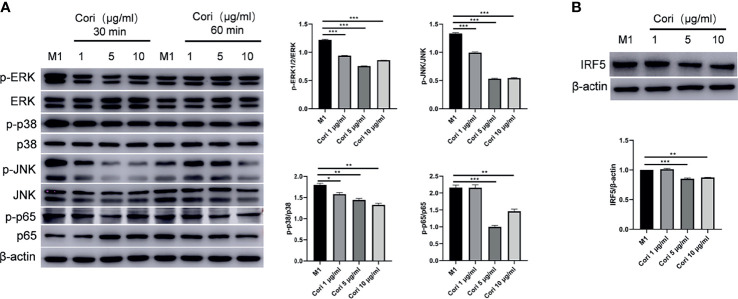
Corilagin inhibits M1 macrophage activation by inhibiting the MAPK, NF-κB and IRF signaling pathways. BMDMs were treated with corilagin (1, 5, or 10 μg/ml) for 2 h, followed by stimulation of LPS and IFN-γ. After 30 and 60 min, cells were collected for the detection of MAPK and NF-κB signaling pathway-related molecules. Finally, 24 h later the cells were collected for the detection of IRF5. **(A)** Western blot analysis and statistics of the phosphorylation levels of JNK, ERK, p38, and NF-κB p65. **(B)** Western blot analysis and statistics for IRF5. *p < 0.05, **p < 0.01, ***p < 0.001.

It has been reported that the MAPK and NF-κB signaling pathways play a key role in the pro-inflammatory response in macrophages (39). Therefore, we examined the activation of these transcription factors or signaling molecules by western blotting. The results showed that corilagin inhibited the phosphorylation of ERK1/2, c-Jun N-terminal kinase (JNK), p38, and p65 after 30 min of treatment (**Figure 6A**). We also examined the expression of IRF5, a key transcription factor for M1 macrophage differentiation. The results indicated that corilagin reduced the expression of IRF5 (**Figure 6B**). Taken together, these results confirm that corilagin affects the MAPK, NF-κB, and IRF signaling pathways, resulting in the inhibition of M1 macrophage activation *in vitro*.

## Discussion

The hepatoprotective effects of corilagin have been reported in liver fibrosis, drug-induced liver damage, and hepatic carcinoma. However, the effects and mechanism of corilagin on immune-mediated hepatic injury have not been fully elucidated. In this study, we investigated the effects of corilagin on Con A-induced hepatic injury. *In vivo* experiments showed that with corilagin treatment reduced ALT and AST levels in the serum and improved histopathological damage, hepatocyte apoptosis, and oxidative stress in the liver. Moreover, the activation of M1 macrophages and expression of M1 macrophage-related inflammatory cytokines and genes were significantly reduced after corilagin treatment. *In vitro* experiments demonstrated the regulation of corilagin on macrophages *via* the MAPK, NF-κB, and IRF signaling pathways.

Oxidative stress plays a vital role in the progression of liver damage in a mouse model of Con A-induced hepatitis. Corilagin has been demonstrated to have anti-oxidative effects (40). ROS can trigger lipid peroxidation, which affects cell structure and function by influencing the fluidity and permeability of the cell membrane. MDA is a marker of the lipid peroxidation reaction on the membrane. Mice challenged with Con A show increased MDA levels in the liver (35, 41). Thus, we measured MDA levels in liver tissue. We found that corilagin treatment reduced MDA levels compared with the Con A group, which is consistent with the findings of previous studies demonstrating that corilagin reduces MDA levels in APAP-induced hepatotoxicity and ischemia/reperfusion-induced acute lung injury (28, 29, 36). In liver tissue, SOD, a hepatoprotective enzyme, is a key antioxidant enzyme produced by Kupffer cells and hepatocytes, which decreases with Con A treatment. In our study, corilagin increased the content of SOD compared with the Con A group, indicating decreased oxidative stress in hepatocytes. MPO is a heme-containing peroxidase that is highly expressed in neutrophils and monocytes and involved in tissue injury in many diseases. Therefore, MPO is regarded as an effective marker of oxidative impairment in neutrophils (42). As previously reported, the MPO level is significantly enhanced with Con A treatment (40); based on our data, corilagin can prevent this progress. These results suggest that corilagin reduces oxidative stress and prevents Con A-induced liver damage.

The Con A-induced hepatitis mouse model is commonly used to investigate immune-mediated liver diseases. The mechanisms underlying the pathological conditions have long been studied and involve multiple cell types and cytokines. NKT, CD4^+^ T, and Kupffer cells are regarded as the main cell types that mediate this process (43). Therefore, we compared the proportion of these cells in the liver between the Con A and corilagin treatment groups. The results revealed that the proportion of CD4^+^ T cells, activated CD4^+^ T cells, NKT cells and macrophages was significantly decreased. This confirmed again that all these cells are involved in the protective effect of corilagin in Con A-induced liver injury. Liver macrophages account for approximately 20% of liver immune cells and play critical roles in Con A-induced hepatitis. To date, the effect of corilagin on immune cells has not been extensively studied, with only a few studies suggesting that corilagin exerts anti-inflammatory effects by regulating the release of inflammatory cytokines in the macrophage cell line (RAW264.7) (44). Therefore, we mainly focused on the role of macrophages in the protective effects of corilagin on Con A-induced hepatitis.

Our data showed that the expression of IL-6, IL-12, TNF-α, and iNOS in serum and liver tissues was markedly reduced after corilagin treatment. The results from our adoptive transfer experiment suggested that ALT and AST levels and the release of inflammatory cytokines were significantly reduced after adoptive transfer with corilagin-treated macrophages, and the histopathological injury also improved. Thus, the data suggest that corilagin can directly regulate macrophages, consequently protecting against Con A-induced liver injury.

Activation of the MAPK, NF-κB, and IRF signaling pathways is a feature of acute inflammatory activation of macrophages (39). Previous studies have shown that corilagin exerts anti-inflammatory and anti-oxidation activities by inhibiting the expression of key molecules associated with the MAPK and NF-κB signaling pathways in APAP-induced liver injury (29). However, they did not investigate the effects of corilagin on immune cells. Other studies have also demonstrated the anti-inflammatory effects of corilagin on multiple cell lines *via* the NF-κB signaling pathway, including the mouse macrophage cell line (RAW264.7) (44) and murine microglial cell line (BV-2) (45). In the present study, we analyzed the effects of corilagin on BMDMs and demonstrated that it can inhibit the polarization of M1 macrophages and reduce the expression of M1 macrophage-related inflammatory cytokines and genes. In addition, corilagin inhibited the phosphorylation of ERK1/2, JNK, p38, and p65 and reduced the expression of IRF5 in BMDMs, suggesting that corilagin can modulate the activation of M1 macrophages by restricting relevant signaling molecules.

In Con A induced liver injury, the binding of Con A to the Kupffer cells (KCs) results in the activation of KCs, which release inflammatory mediators. The MHC class II on KCs modified by Con A could be recognized by T cell receptors on T cells, and make T cells activated, thereby enhancing the secretion of inflammatory factors, and ultimately leading to liver cell apoptosis (10). Based on our results that corilagin inhibited the activation of macrophages, and it also influenced the activation of T cells and NKT cells, suggesting that corilagin could disrupt the interactions between immune cells in liver injury. Our future studies will focus on the effects of corilagin on macrophage and T cells to address how corilagin affect immune cells network.

**Figure 7:**
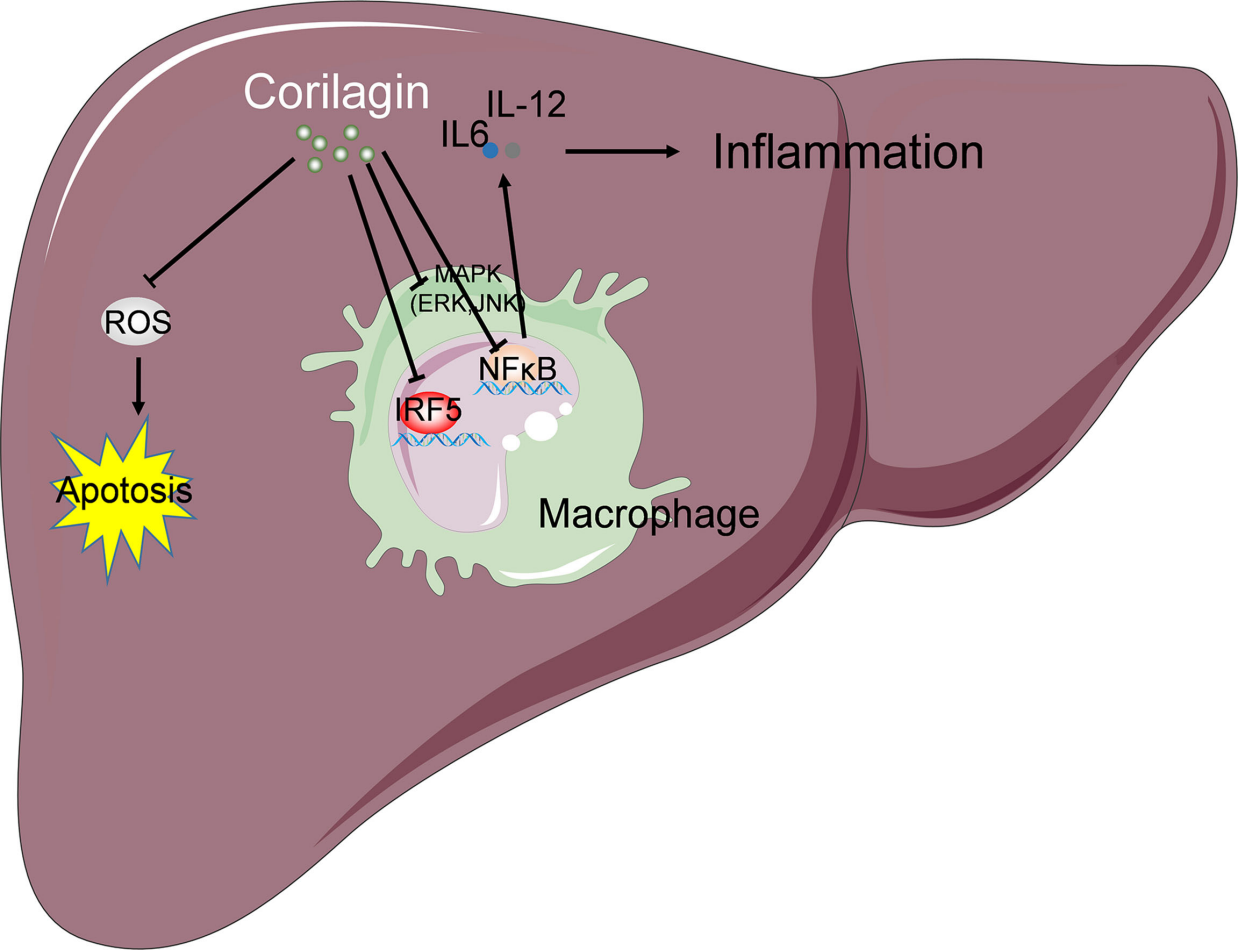
Schematic summary of possible mechanism underlying the protective effects of corilagin on Con A induced liver injury. The mechanism is associated with the inhibition of corilagin on the activation of M1 macrophage *via* MAPK, NF-κB, and IRF signaling pathways, resulting in the suppression of inflammation. Corilagin also attenuates Con A-induced oxidative stress and hepatocyte apoptosis in the liver tissue. “→” indicates promotion or positive regulation; “⊥” indicates inhibition or negative regulation.

In summary, the findings of this study suggest that corilagin exerts a hepatoprotective effect on liver injury induced by Con A treatment. The mechanism is associated with the inhibition of corilagin on the activation of M1 macrophage *via* MAPK, NF-κB, and IRF signaling pathways, resulting in the suppression of inflammation, oxidative stress, and apoptosis in the liver tissue (**Figure 7**). As Con A-induced hepatic injury murine model is the most commonly used for clinical autoimmune hepatitis (AIH), which is a chronic progressive inflammation of liver parenchyma mediated by autoimmune response (46, 47). Although AIH is still rare disease, its occurrence is widespread worldwide and the incidence and prevalence are increasing (48). If AIH cannot be treated in time, it can lead to cirrhosis and liver failure. The cornerstone of treatment is steroid induction therapy followed by maintenance therapy with azathioprine (48). One study found that relapse of the disease occurs in up to 90% of patients once treatment is stopped (49). It has not been reported that corilagin is clinically used to treat clinical autoimmune hepatitis. Thus, our results may provide novel evidence for the future therapeutic applications of corilagin in immune-mediated hepatic injury.

## Data Availability Statement

The original contributions presented in the study are included in the article/**Supplementary Material**. Further inquiries can be directed to the corresponding authors.

## Ethics Statement

The animal study was reviewed and approved by Animal Care Committee of Jining Medical University.

## Author Contributions

FY, DC, HW, MG, and HC performed the experiments. FY, HZ, JZ, and CW analyzed the data and generated figures. FY, HZ, and HX wrote the manuscript. All authors contributed to the article and approved the submitted version.

## Funding

This work was supported by the National Natural Science Foundation of China (No. 81874169, 81801557 and 82171810), Projects of Medical and Health Technology Development Program in Shandong Province, China (No. 202002070949), NSFC cultivation project of Jining Medical University (No. JYP2018KJ22), Natural Science Foundation of Shandong Province (No. ZR2021MH287) and Shandong Training Program of Innovation and Entrepreneurship for Undergraduates (No. S202110443039).

## Conflict of Interest

The authors declare that the research was conducted in the absence of any commercial or financial relationships that could be construed as a potential conflict of interest.

## Publisher’s Note

All claims expressed in this article are solely those of the authors and do not necessarily represent those of their affiliated organizations, or those of the publisher, the editors and the reviewers. Any product that may be evaluated in this article, or claim that may be made by its manufacturer, is not guaranteed or endorsed by the publisher.
