# Supplementary material for: Corilagin Ameliorates Con A-Induced Hepatic Injury by Restricting M1 Macrophage Polarization
Source: Front Immunol. 2022 Jan 13;12:807509. doi: 10.3389/fimmu.2021.807509 (PMC8792905; doi:10.3389/fimmu.2021.807509)
Supplement: Supplementary file 1 [file DataSheet_1.zip › Supplementary_Material-corrected/Supplementary_Material-corrected.docx]

Supplementary Material

## Supplementary Figures


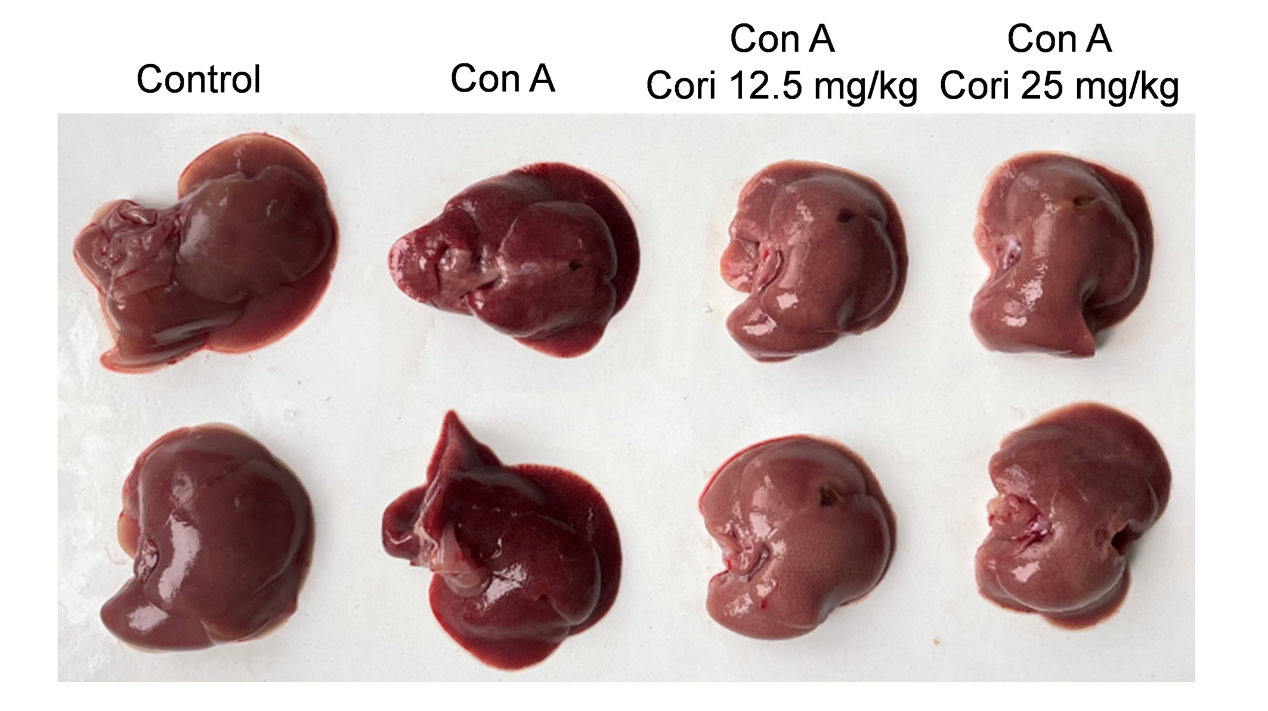


**Supplementary Figure 1.** Liver phenotype with different concentrations of corilagin treatment. Mice were administered with corilagin (12.5, 25 mg/kg) intraperitoneally twice at time intervals of 12 h. After 1 h, mice were challenged with Con A (20 mg/kg body weight). After 12 h, livers were collected and photographed.


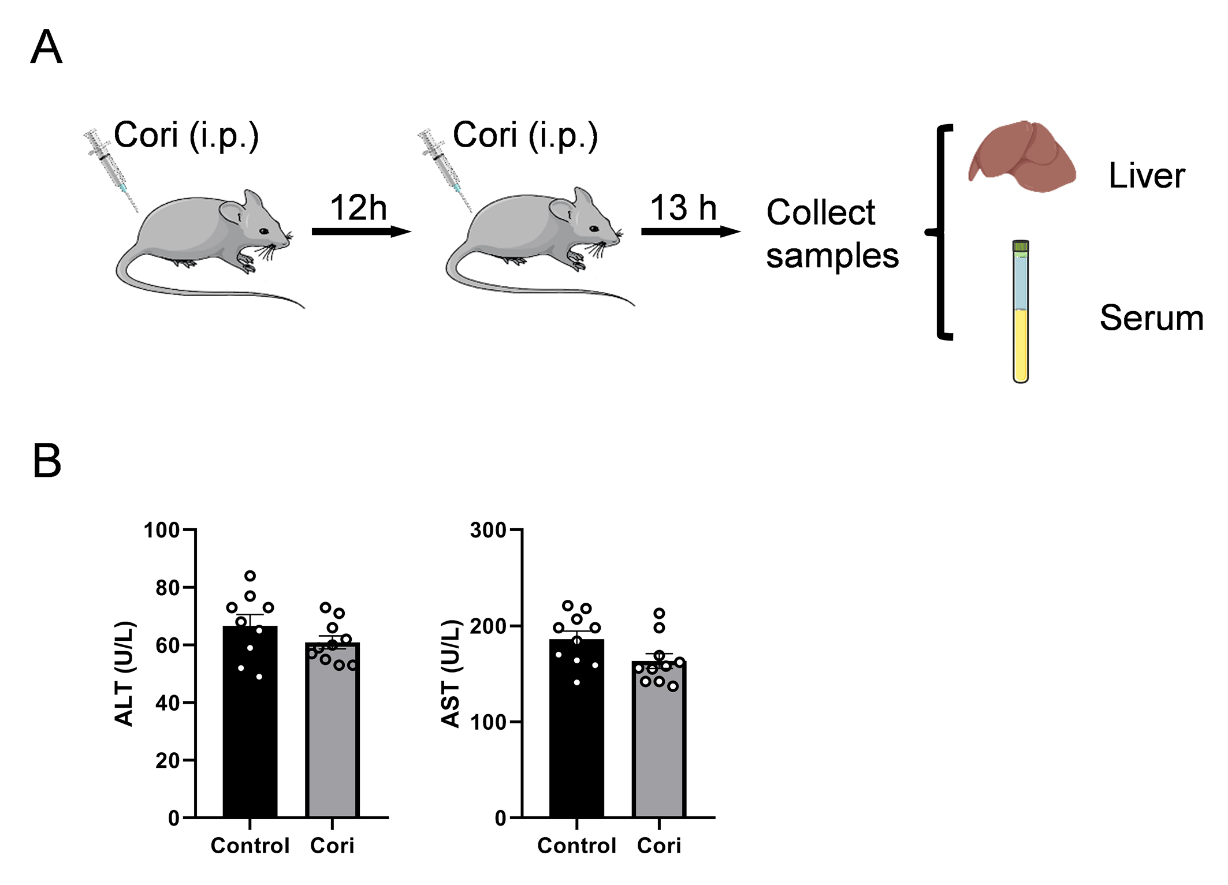


**Supplementary Figure 2.** Corilagin treatment alone have no effect on the liver. Mice were administered corilagin (25 mg/kg body weight) intraperitoneally twice at time intervals of 12 h. After 13 h, serum and liver samples were collected (n=10). A. The flowchart for corilagin administration. B. Serum alanine aminotransferase (ALT) and aspartate aminotransferase (AST) levels.


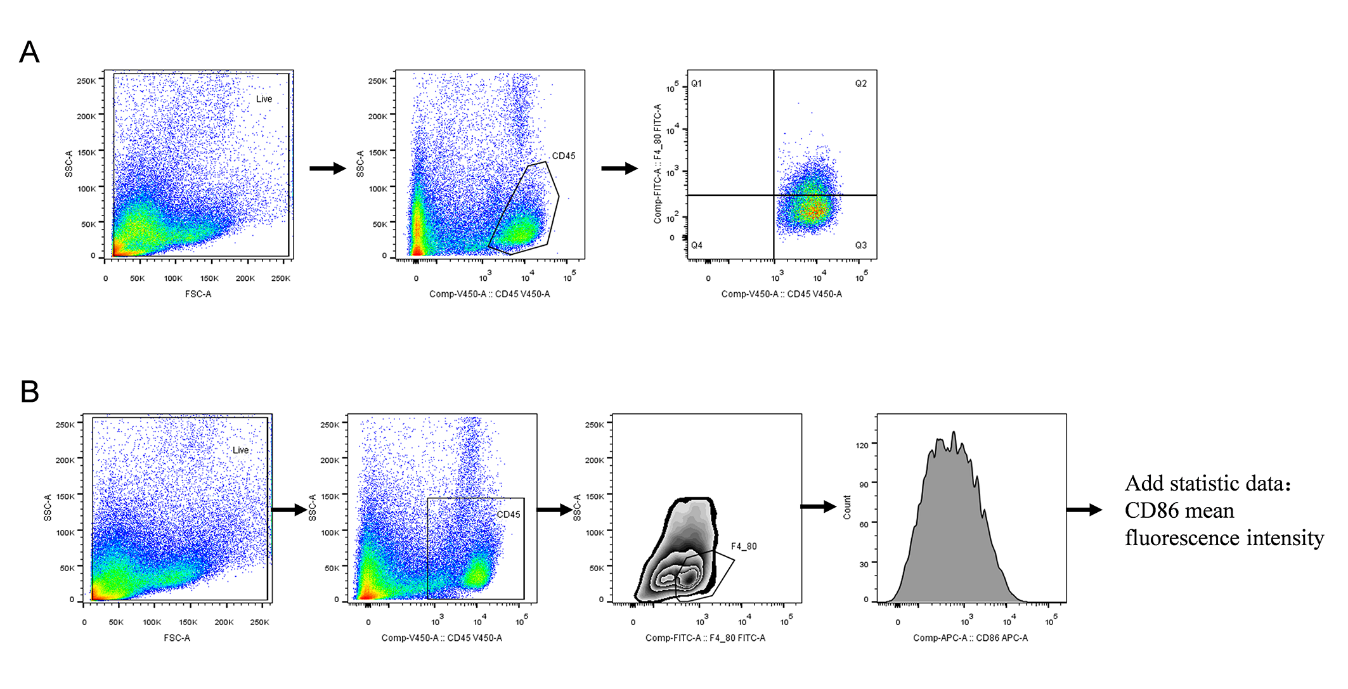


**Supplementary Figure 3.** **A.** Detail gating strategy for Figure 3A. **B.** Detail gating strategy for Figure 3B.

**
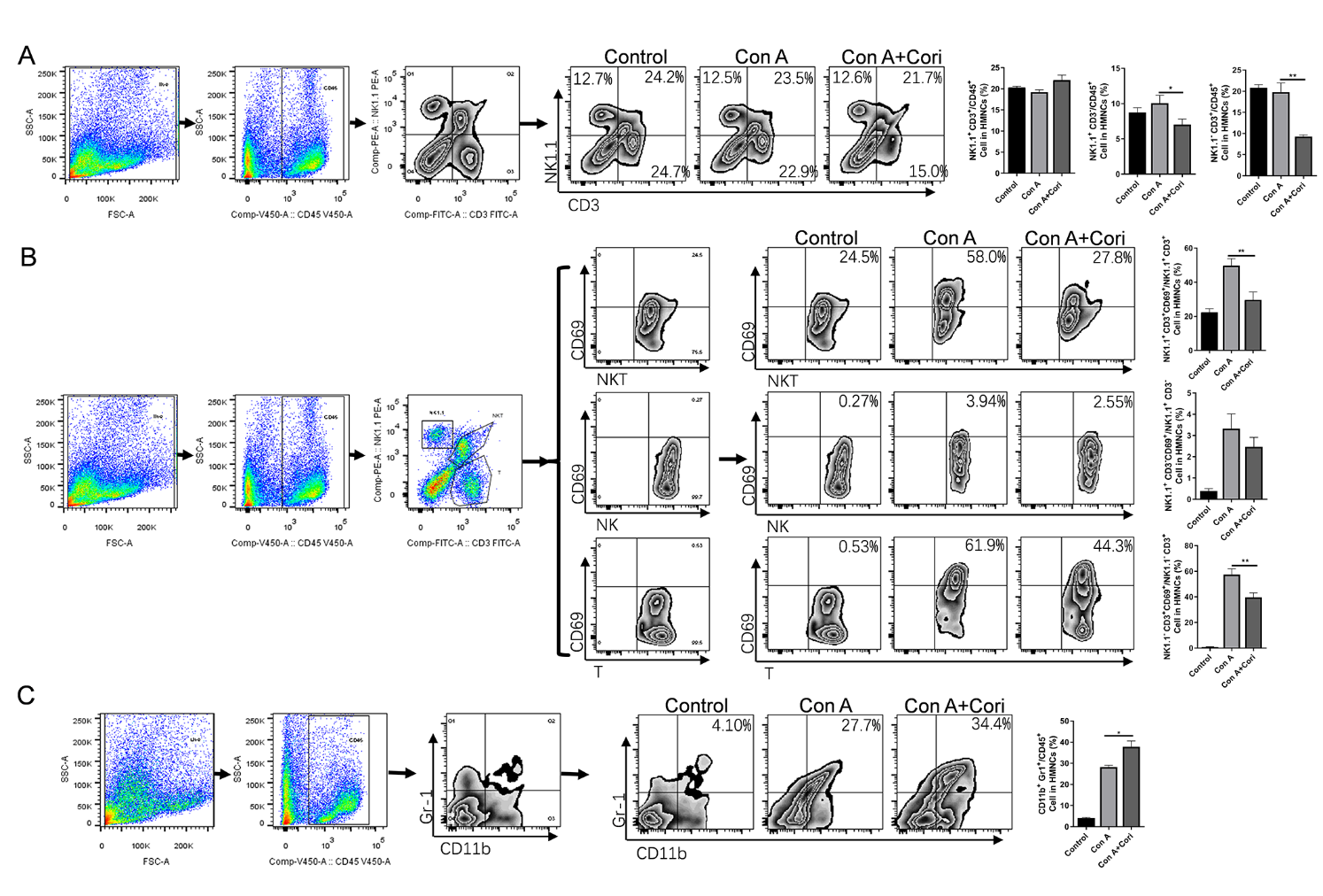
**

**Supplementary Figure 4.** The effects of corilagin on NK, NKT, T and MDSCs in liver. Mice were administered corilagin (25 mg/kg) intraperitoneally twice at time intervals of 12 h. After 1 h, mice were challenged with Con A (20 mg/kg body weight). liver tissues were collected 12 h later. **A.** Detection of the NKT (CD3^+^NK1.1^+^), NK (CD3^-^NK1.1^+^), and T (CD3^+^NK1.1^-^) cells ratio in mouse hepatic mononuclear cells (HMNCs) by flow cytometry. **B.** The activation of NKT (CD3^+^NK1.1^+^CD69^+^), NK (CD3^-^NK1.1^+^CD69^+^), and T (CD3^+^NK1.1^-^ CD69^+^) cells in mouse HMNCs. **C.** Detection of the MDSCs (CD11b^+^Gr-1^+^) ratio in mouse HMNCs. **p* < 0.05, ***p* < 0.01.

**
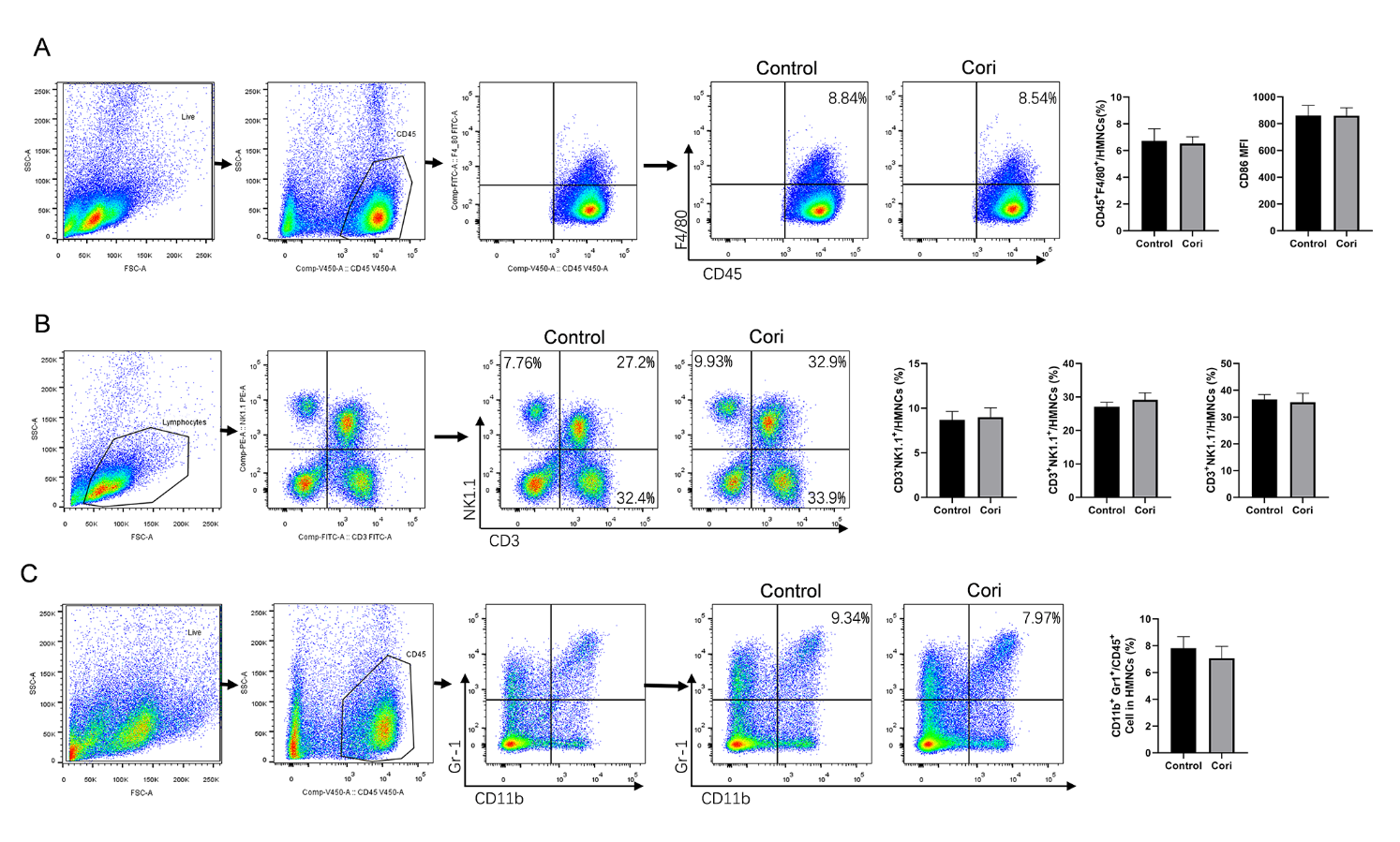
**

**Supplementary Figure 5.** The effects of corilagin treatment alone on NK, NKT, T and MDSCs in liver. Mice were treatment as indicated in supplementary Figure 2. **A.** Detection of the macrophage ratio and CD86 mean fluorescence intensity (MFI) in mouse hepatic mononuclear cells (HMNCs) by flow cytometry. **B.** Detection of the NKT (CD3^+^NK1.1^+^), NK (CD3^-^NK1.1^+^), and T (CD3^+^NK1.1^-^) cells ratio in mouse hepatic mononuclear cells (HMNCs) by flow cytometry. **C.** Detection of the MDSCs (CD11b^+^Gr-1^+^) ratio in mouse HMNCs.

**
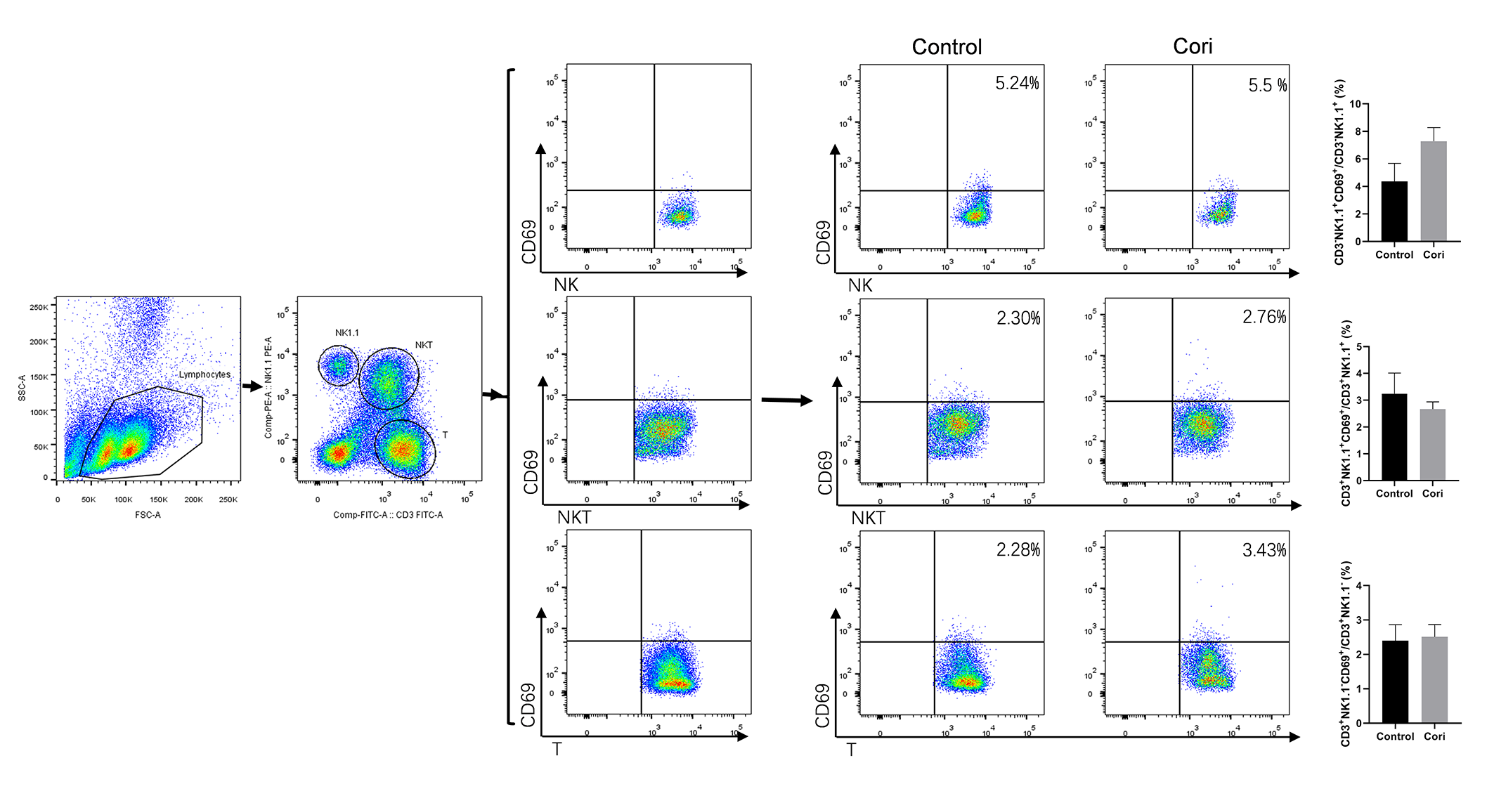
**

**Supplementary Figure 6.** The effects of corilagin treatment alone on the activation of NKT (CD3^+^NK1.1^+^CD69^+^), NK (CD3^-^NK1.1^+^CD69^+^), and T (CD3^+^NK1.1^-^ CD69^+^) cells in mouse HMNCs. Mice were treatment as indicated in supplementary Figure 2.


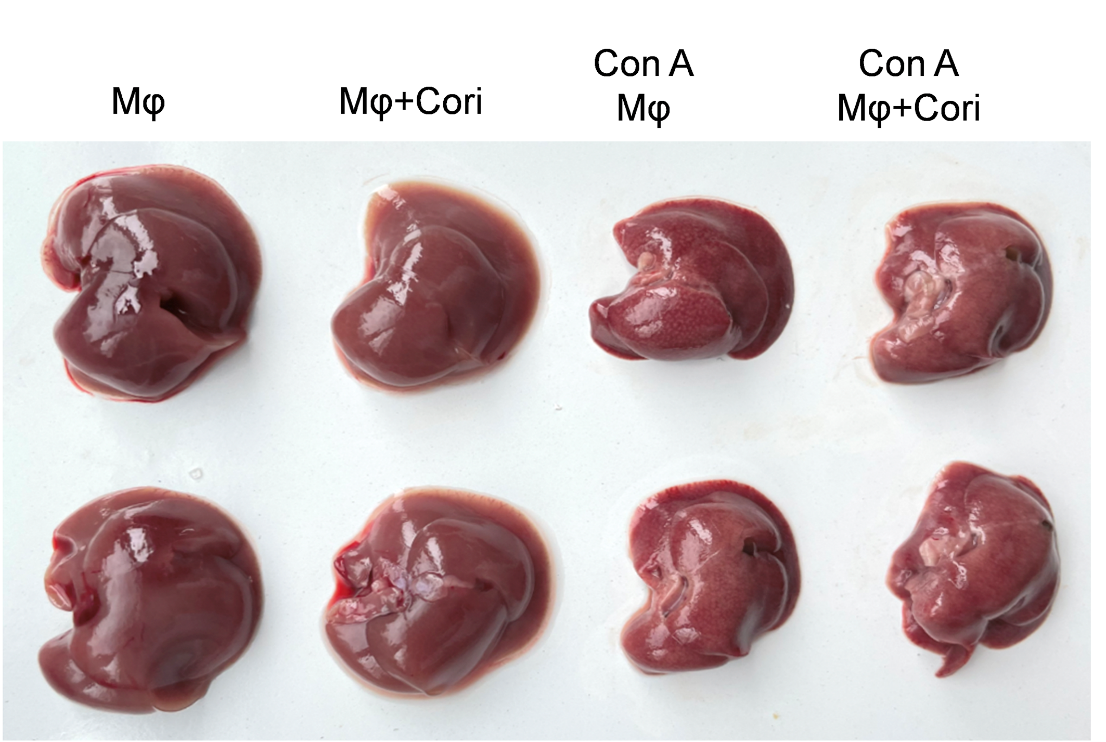


**Supplementary Figure 7.**  Liver phenotype of adoptiver transfer experiment. Macrophage cultured *in vitro* were treated with corilagin (10 μg/ml); 3 h later these cells were collected and injected into mice intraperitoneally. After 12 h, mice were challenged with Con A (20 mg/kg body weight). Liver tissues were collected and photographed 12 h later.

**
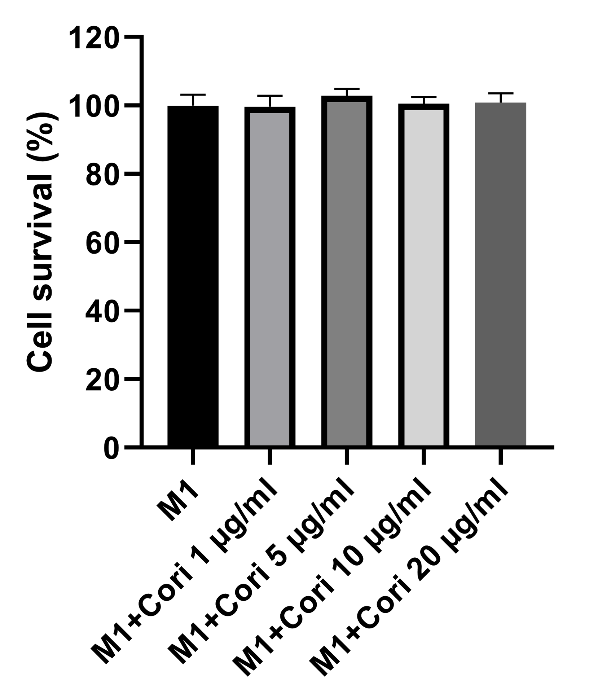
**

**Supplementary Figure 8.** Corilagin had no cytotoxicity on macrophage. BMDMs were treated with corilagin (1, 5, 10, or 20 μg/ml) for 2 h, followed by stimulation with LPS and IFN-γ. 24 hours later, the cytotoxicity of corilagin on macrophage were detected with CellTiter-Lumi Luminescent Cell Viability Assay Kit.

**
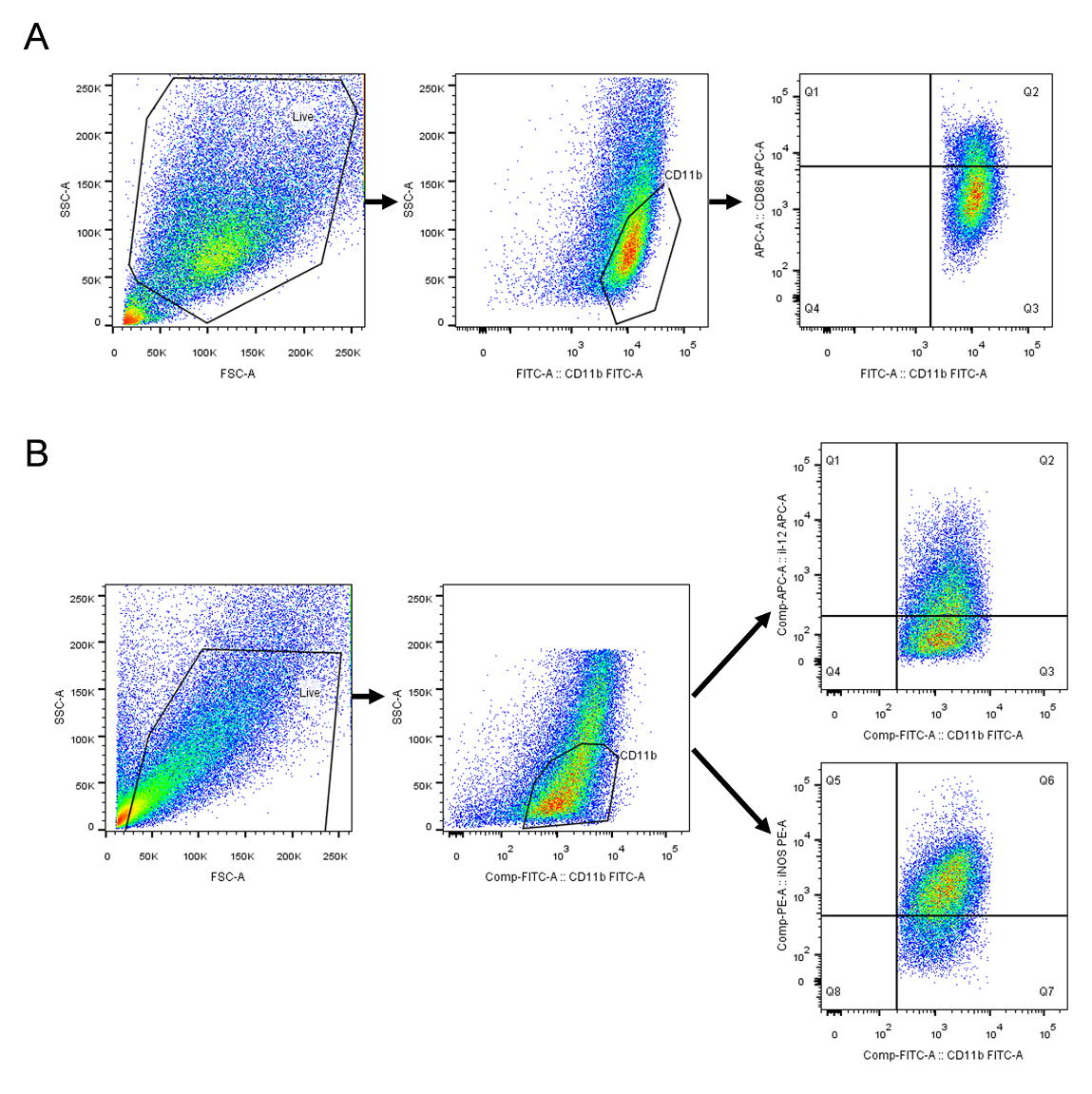
**

**Supplementary Figure 9.** Detail gating strategy for Figure 5A, 5B and 5C. **A.** Detail gating strategy for Figure 5A. **B.** Detail gating strategy for Figure 5B and 5C
