# Supplementary figures and images for: Corilagin Ameliorates Con A-Induced Hepatic Injury by Restricting M1 Macrophage Polarization
Source: Front Immunol. 2022 Jan 13;12:807509. doi: 10.3389/fimmu.2021.807509 (PMC8792905; doi:10.3389/fimmu.2021.807509)

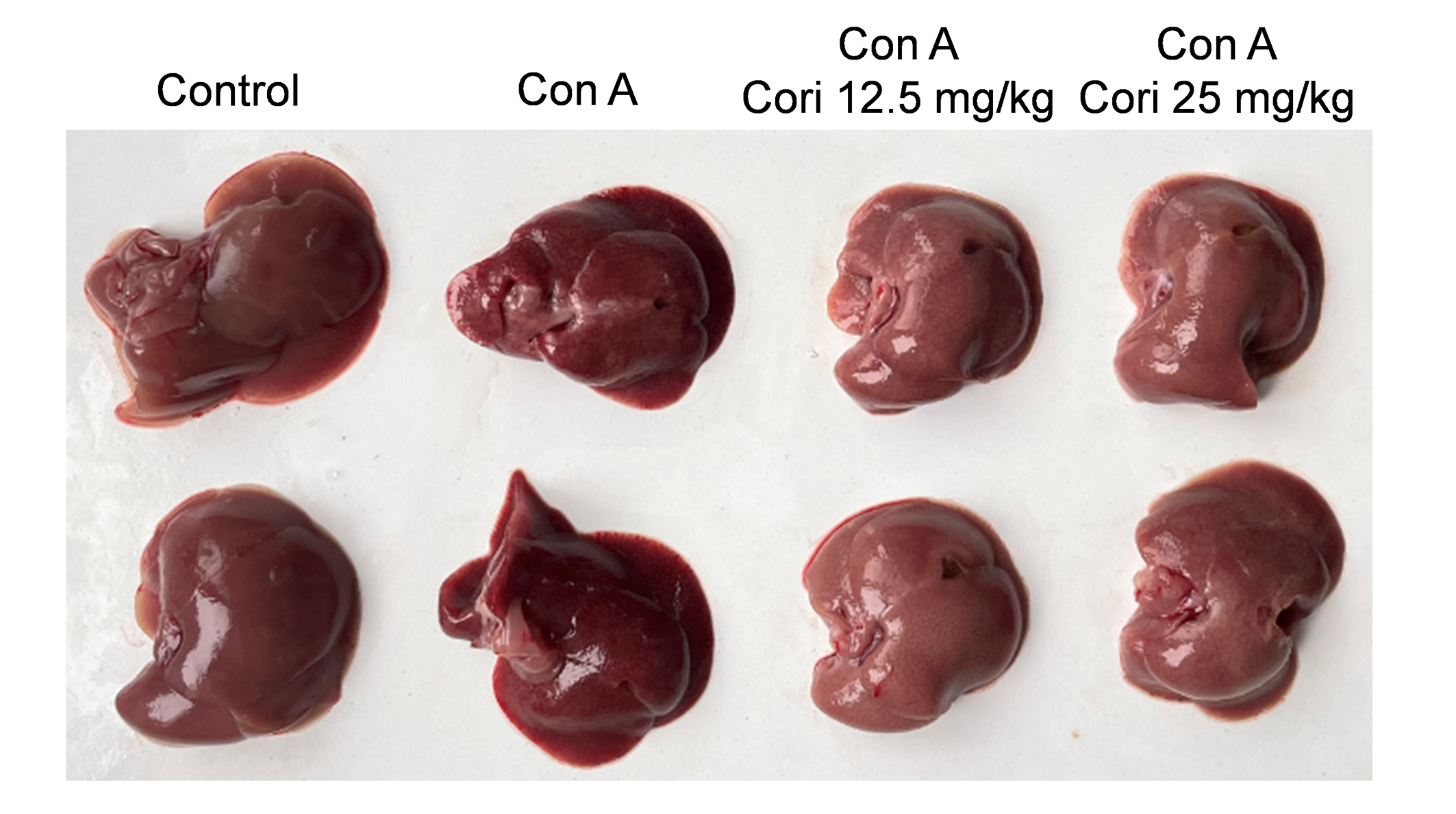

Supplement: Supplementary file 1 [file DataSheet_1.zip › Supplementary_Material-corrected/Supplementary figure 1.tif]

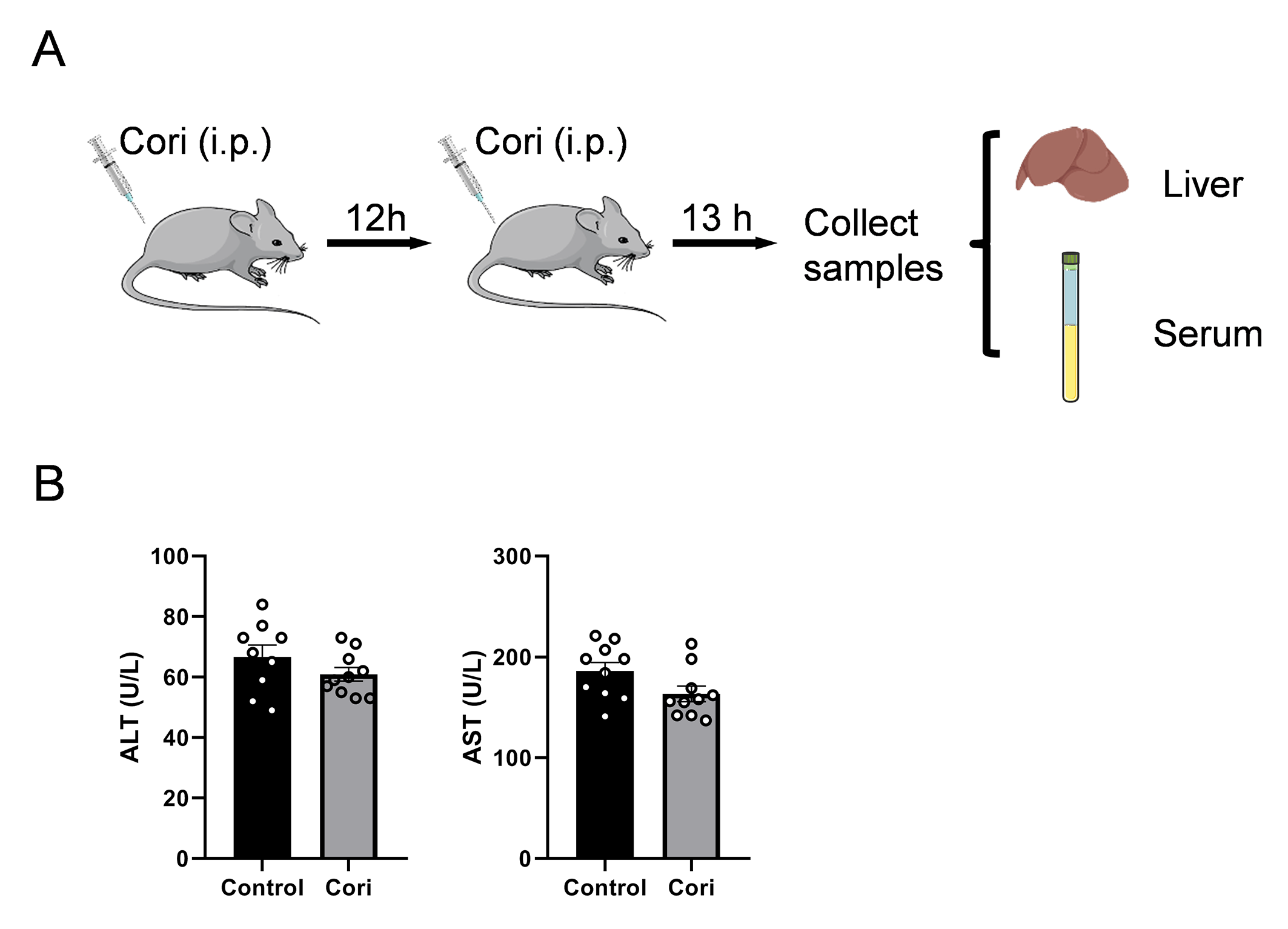

Supplement: Supplementary file 1 [file DataSheet_1.zip › Supplementary_Material-corrected/Supplementary figure 2.tif]

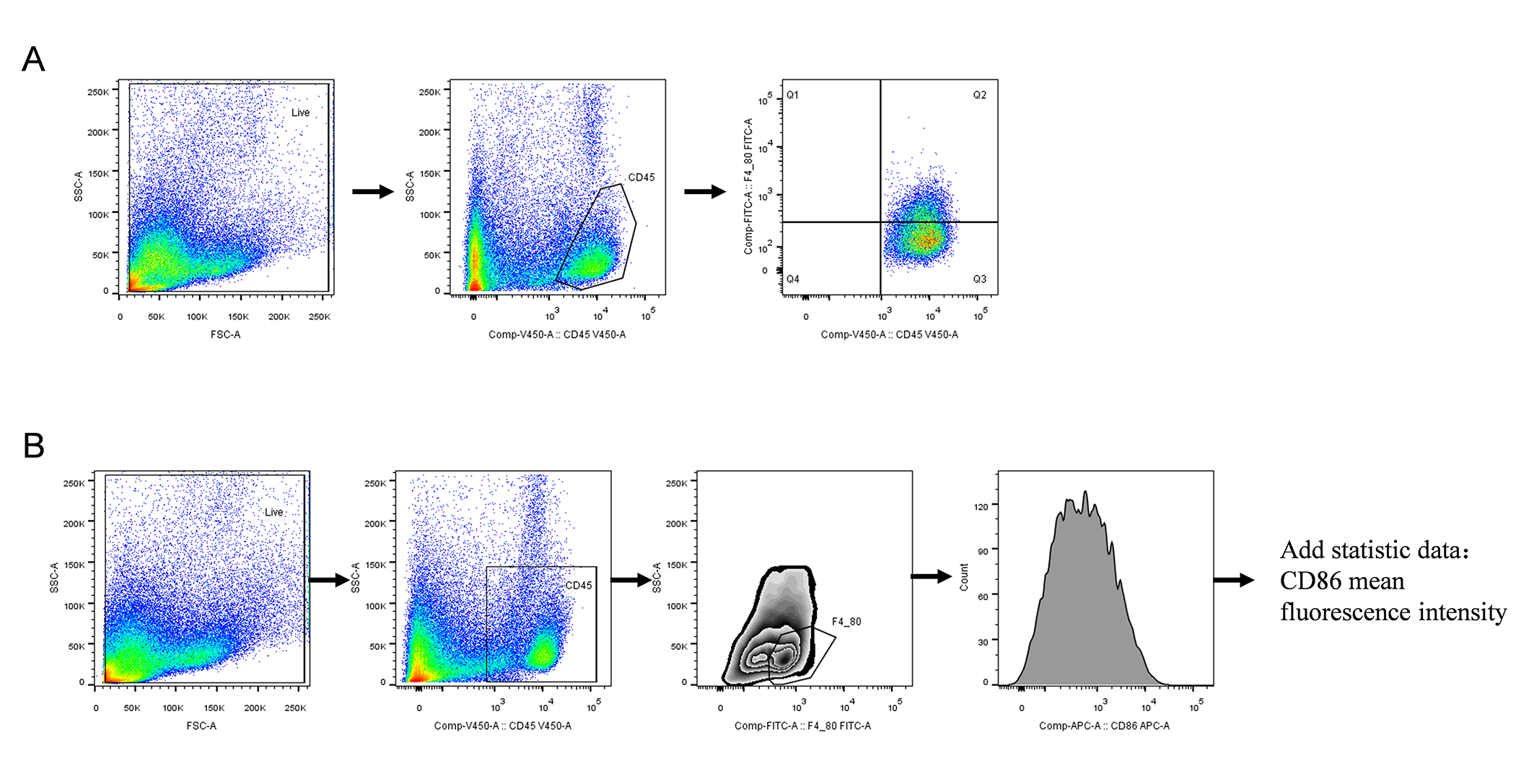

Supplement: Supplementary file 1 [file DataSheet_1.zip › Supplementary_Material-corrected/Supplementary figure 3.tif]

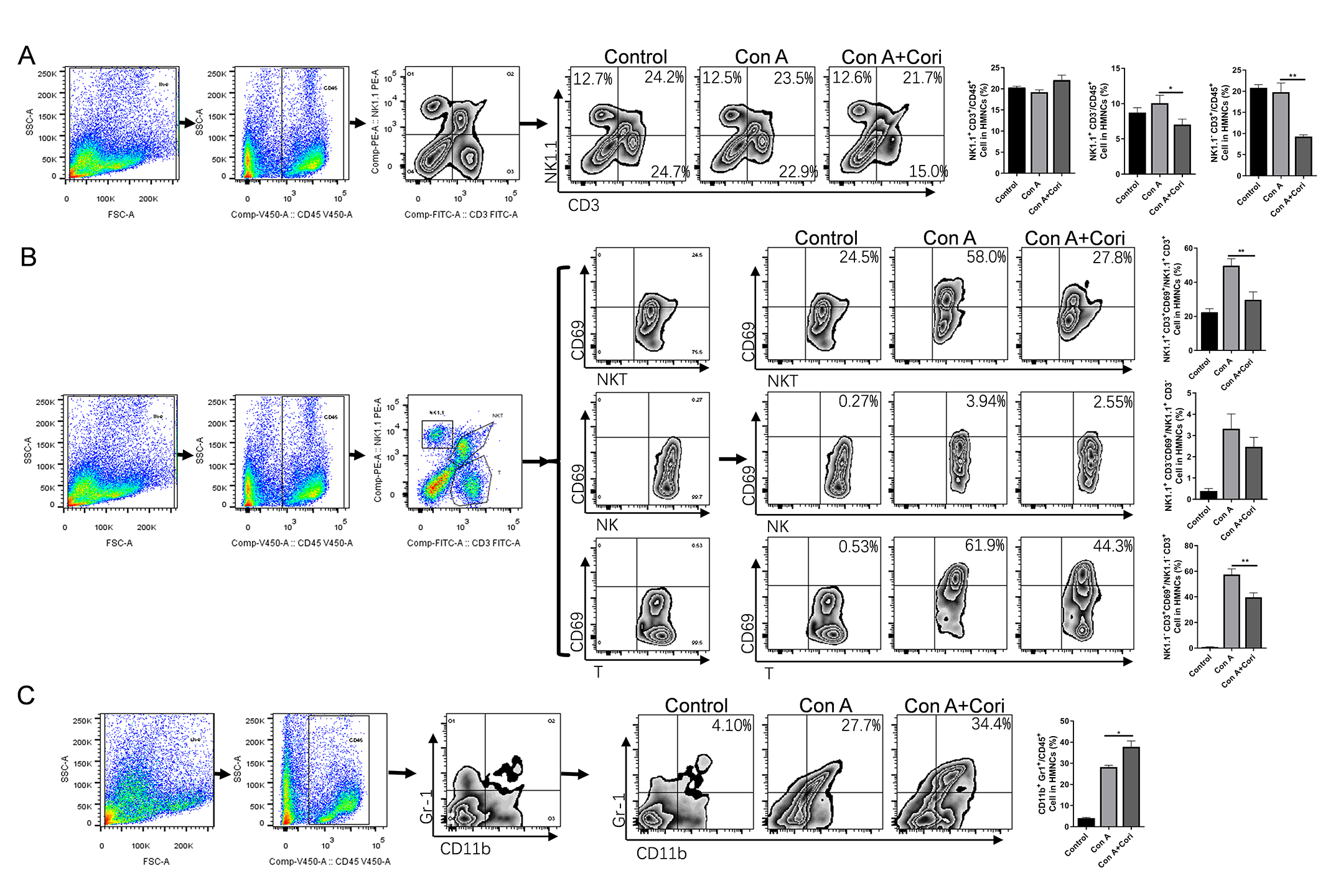

Supplement: Supplementary file 1 [file DataSheet_1.zip › Supplementary_Material-corrected/Supplementary figure 4.tif]

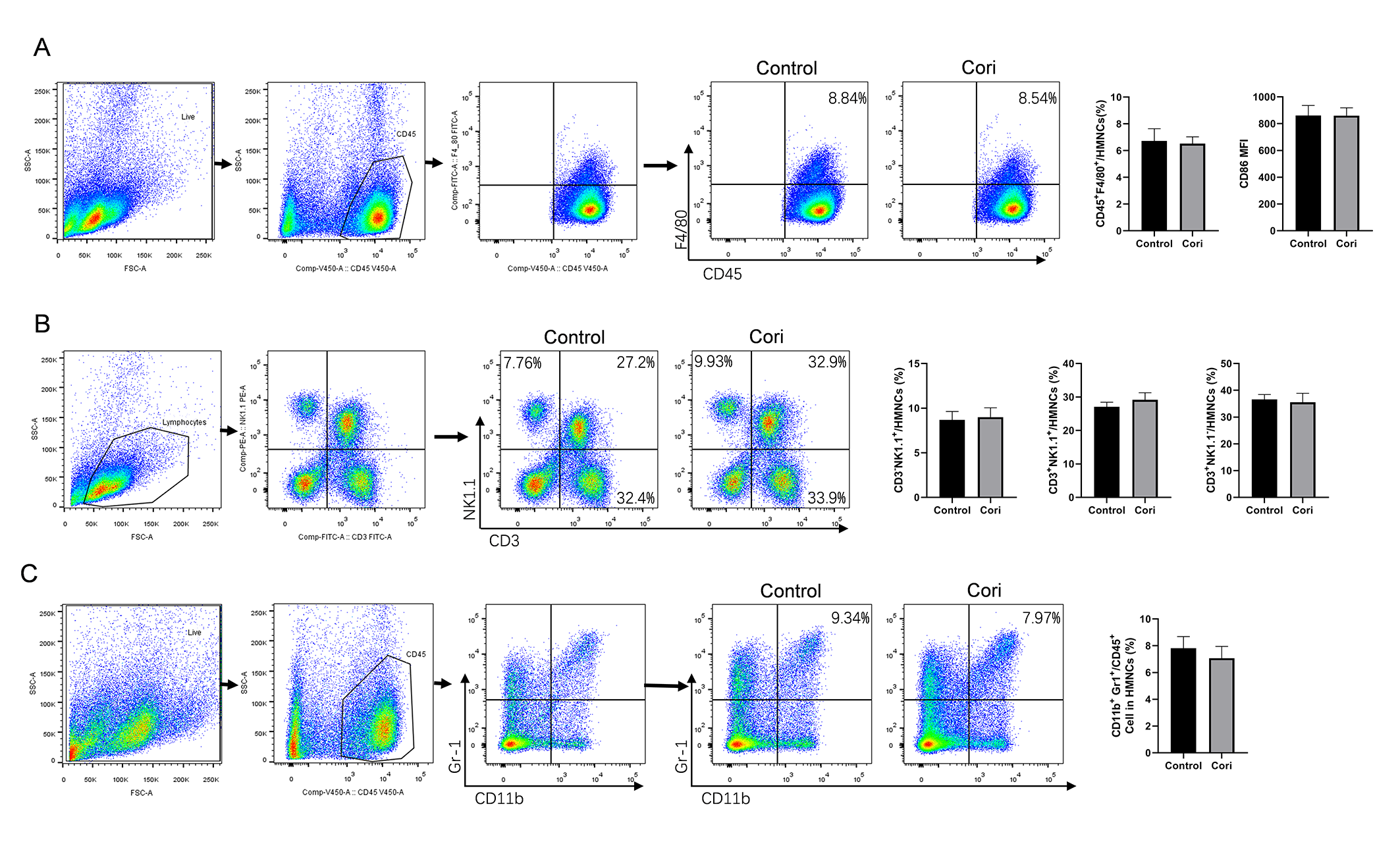

Supplement: Supplementary file 1 [file DataSheet_1.zip › Supplementary_Material-corrected/Supplementary figure 5.tif]

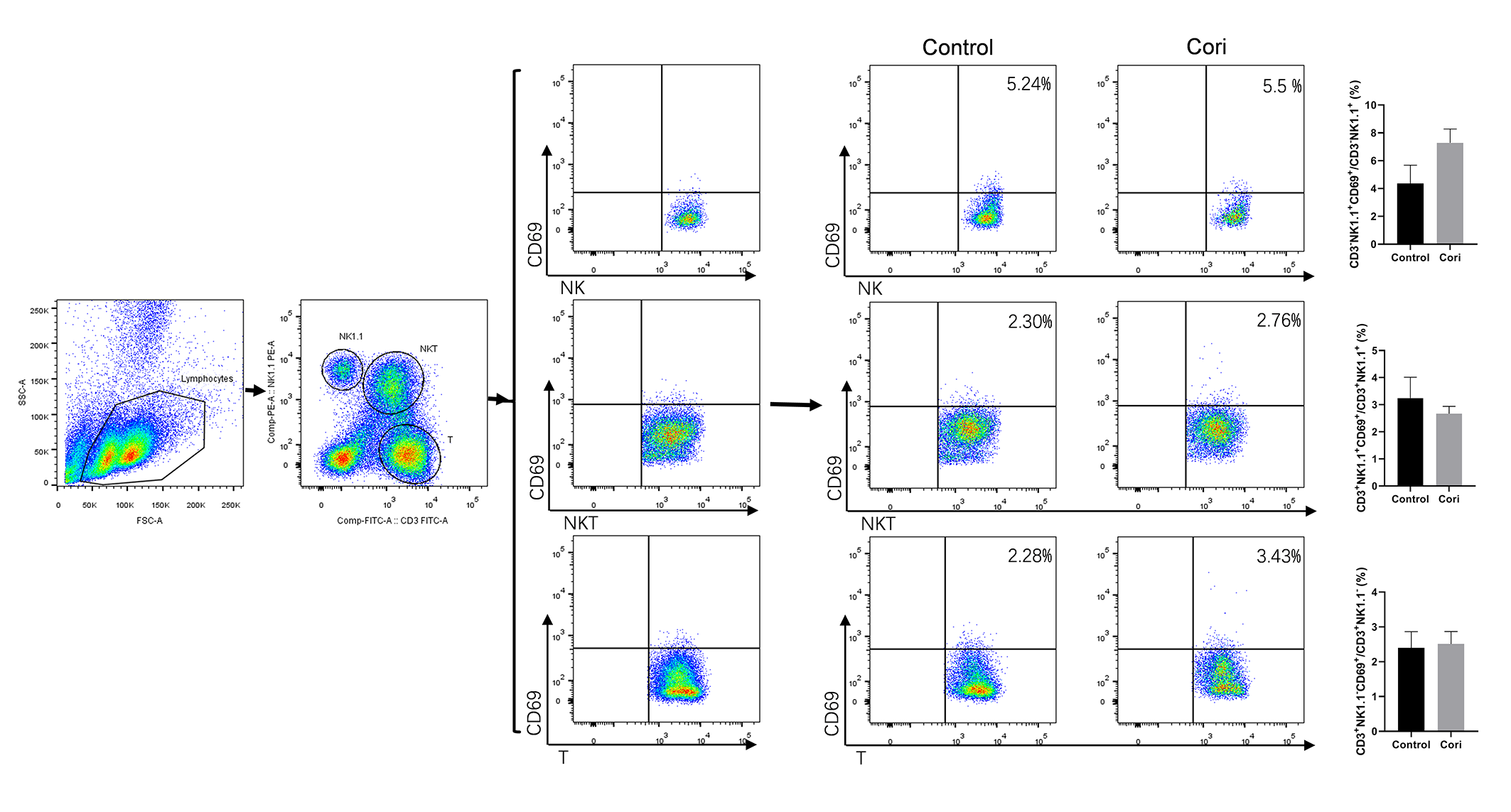

Supplement: Supplementary file 1 [file DataSheet_1.zip › Supplementary_Material-corrected/Supplementary figure 6.tif]

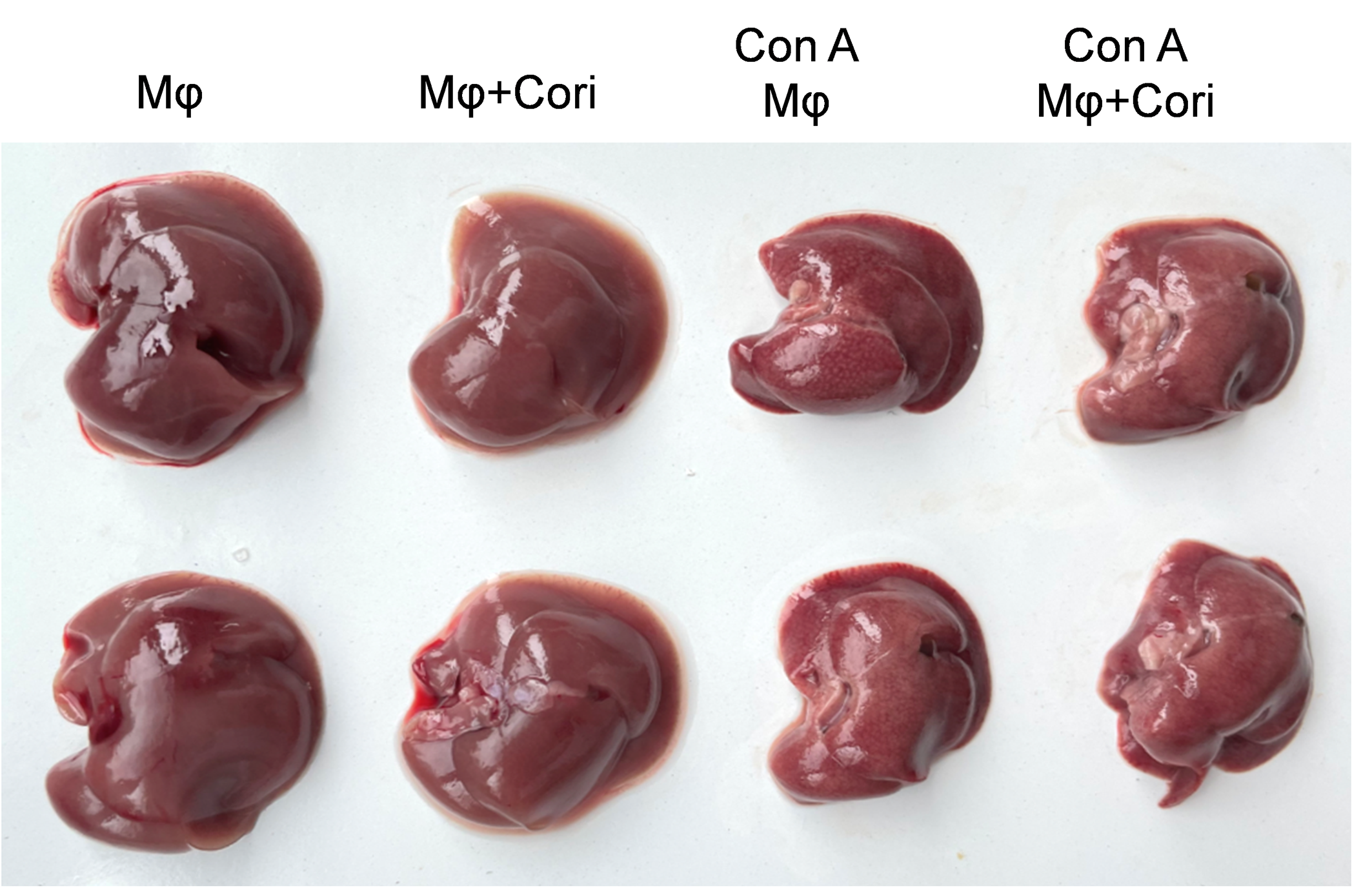

Supplement: Supplementary file 1 [file DataSheet_1.zip › Supplementary_Material-corrected/Supplementary figure 7.tif]

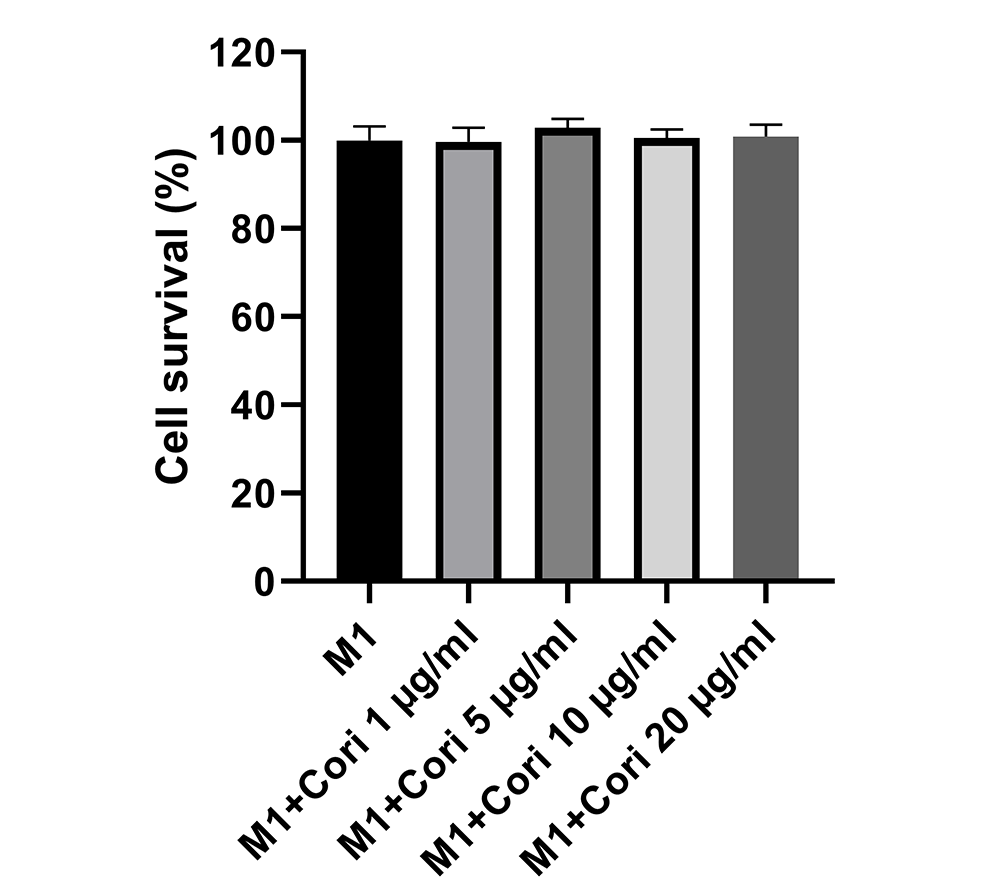

Supplement: Supplementary file 1 [file DataSheet_1.zip › Supplementary_Material-corrected/Supplementary figure 8.tif]

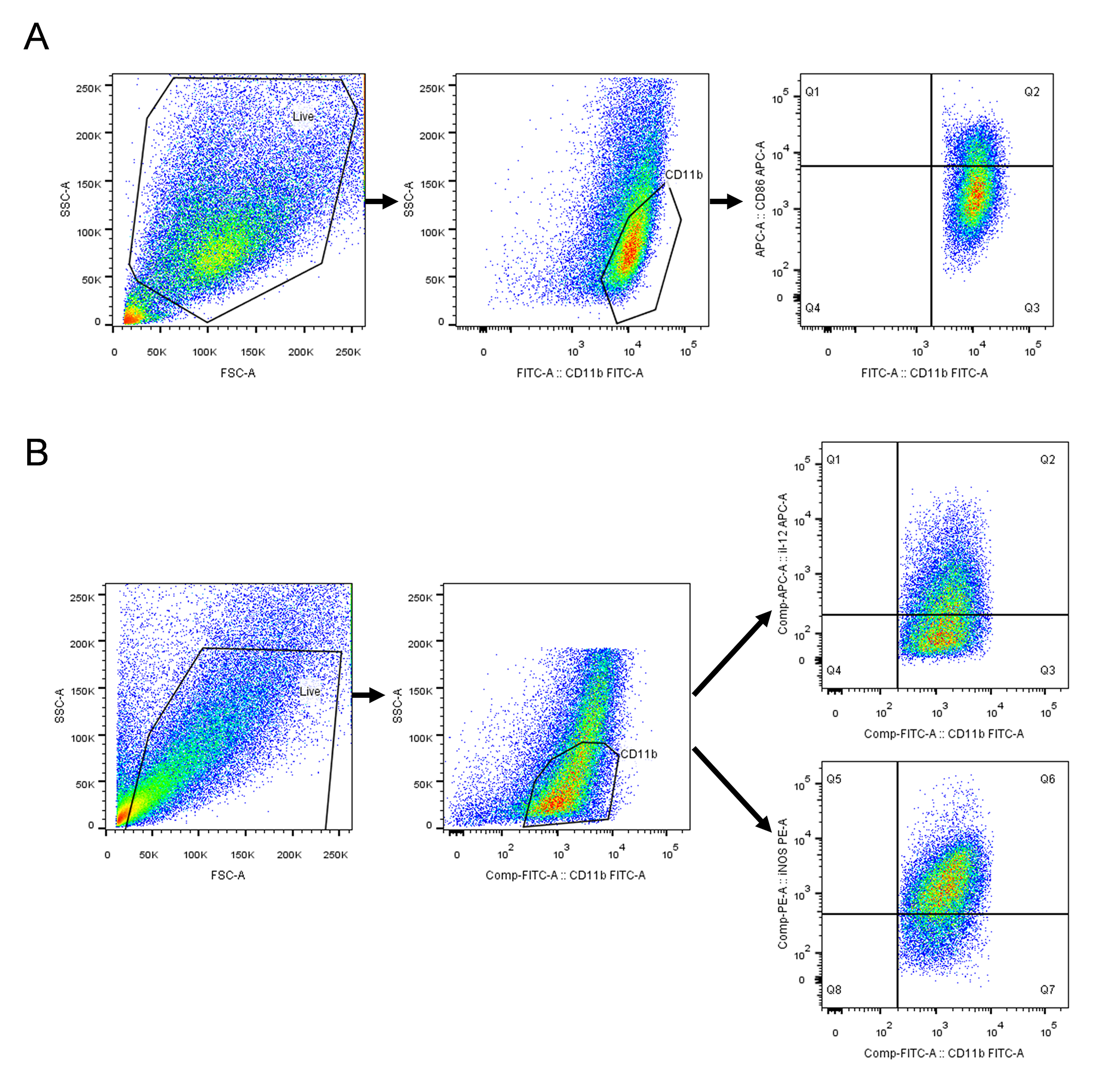

Supplement: Supplementary file 1 [file DataSheet_1.zip › Supplementary_Material-corrected/Supplementary figure 9.tif]
